# Neurobiological basis of suicidal behavior: a narrative review of cerebrospinal fluid monoamine metabolites

**DOI:** 10.3389/fpsyt.2026.1968256

**Published:** 2026-10-19

**Authors:** Lucrezia Stefani, Luigi Tonino Marsella, Roberta Tittarelli

**Affiliations:** Laboratory of Forensic Toxicology, Section of Forensic Medicine and Toxicology, Department of Biomedicine and Prevention, University of Rome Tor Vergata, Rome, Italy

**Keywords:** 5-HIAA (5-hydroxyindoleacetic acid), biomarkers, cerebrospinal fluid - CSF, dopamine, HVA, serotonin, suicidal behavior (SB), suicide

## Abstract

**Background:**

Suicide is a multifactorial phenomenon resulting from the interaction of psychiatric, biological, genetic, psychological, environmental and sociocultural factors. Evidence suggests that alterations in central neurotransmitter systems may contribute to vulnerability to suicidal ideation. Among the biological markers investigated, cerebrospinal fluid (CSF) concentrations of 5-hydroxyindoleacetic acid (5-HIAA) and homovanillic acid (HVA), have received particular attention.

**Methods:**

A literature review was conducted using Medline/PubMed and Google Scholar to identify studies published between 2005 and 2026 addressing suicidal behavior, suicide attempts, lethality, and their biological and clinical correlates, and 5-HIAA and HVA, with particular emphasis on their CSF concentrations. Studies published in languages other than English and review articles were excluded. Studies investigating 5-HIAA and HVA in other biological matrices were considered when they provided relevant contextual evidence for the interpretation of suicidal behavior. A total of 23 studies were included in the review.

**Results:**

Evidence supports an association between suicidal behavior and alterations in central monoaminergic neurotransmission. Reduced CSF 5-HIAA concentrations were the most consistent finding, particularly in mood disorders and violent or high-lethality suicidal behavior. Lower 5-HIAA levels were associated with greater lethality of suicide attempts, suggesting a relationship between impaired serotonergic turnover, behavioral disinhibition, impulsivity, and the severity of suicidal acts. CSF HVA concentrations were also reduced in some populations, although findings were more heterogeneous and less consistent than those observed for 5-HIAA. Studies suggested that monoaminergic abnormalities may be more strongly related to the severity or lethality of suicidal behavior than to its occurrence alone. The neurobiological substrate of suicidality may involve interactions between monoaminergic, inflammatory, metabolic, and neuroendocrine pathways rather than isolated biomarkers.

**Conclusions:**

Evidence supports the relevance of CSF monoamine metabolites, particularly 5-HIAA and, to a lesser extent, HVA, as biological correlates of suicidal behavior. However, heterogeneity across psychiatric diagnoses, small sample sizes, differences in CSF collection and analytical procedures, and predominantly cross-sectional study designs limit the generalizability and clinical translation of these findings. The invasive nature of lumbar puncture makes CSF biomarkers unsuitable for routine suicide-risk screening currently. Further longitudinal research is needed to determine whether their integration with clinical and peripheral biological measures could contribute to objective risk stratification and more personalized suicide prevention strategies.

## 1 Introduction

Globally, suicide accounts for more than 1% of all deaths, with an average mortality rate of nearly 10 per 100,000 population, corresponding to approximately one suicide every 40 seconds (1). In 2021, an estimated 727,000 people died by suicide worldwide, representing 1.1% of all deaths and underscoring its major public health relevance, with mortality exceeding that attributable to malaria, HIV/AIDS, breast cancer, armed conflict, or homicide. The global age-standardized suicide rate was 8.9 per 100,000 population, although substantial geographical variability persists, with rates ranging from less than 1 to nearly 40 per 100,000 across countries, reflecting differences in socioeconomic conditions, cultural contexts, mental health service availability, reporting systems, and access to lethal means. A marked gender disparity is consistently observed, with higher rates among males (12.3 per 100,000) compared with females (5.6 per 100,000), corresponding to a global male-to-female ratio of approximately 2.2. Notably, a substantial proportion of suicides (73%) occurs in low- and middle-income countries, where mental health infrastructure remains insufficient, contributing to significant gaps in epidemiological knowledge and prevention strategies (2). The true burden of suicide is likely underestimated due to stigma, misclassification of causes of death, and limitations in mortality surveillance systems (3). Age-specific analyses indicate that more than half (56%) of suicide deaths occur in individuals under 50 years, highlighting substantial premature mortality and socioeconomic impact. Suicide is also a leading cause of death among young people, ranking third globally among individuals aged between 15 and 29 years and second among females in this age group, with 84% of adolescent suicides (10–19 years) occurring in low- and middle-income countries, emphasizing the disproportionate burden in resource-limited settings. These epidemiological differences reflect the multifactorial nature of suicidal ideation and the influence of biological, psychological, social, and environmental determinants (4). Suicide is a multifactorial phenomenon shaped by complex and interrelated social, economic, cultural, and psychological determinants. Rather than being attributable to a single cause, suicidal ideation typically emerges from the dynamic interaction between individual vulnerability and adverse environmental conditions; structural factors play a critical role. At the individual level, stressful life events frequently act as precipitating factors. These may include loss of livelihood, unemployment, academic pressure, relationship breakdown, discrimination, and other acute life crises. Such stressors, particularly when combined with underlying mental health conditions or limited social support, can significantly increase the vulnerability to suicidal behavior. Economic insecurity, poverty, and widening inequalities further compound risk, particularly in contexts where social safety nets are weak (5). In some countries, suicide attempts have historically been subject to criminal or other legal penalties (6). The legal status of attempted suicide varies substantially across jurisdictions, and criminalization has been discussed in relation to stigma, help-seeking, and the reporting of suicide attempts (7). This is particularly relevant given the high ratio of suicide attempts to deaths by suicide, with current WHO estimates indicating that there are likely more than 20 suicide attempts for every suicide, although this ratio varies across countries, regions, sex, age, and methods (8). Engaging in one or more acts of attempted suicide or self-harm is the single most important predictor of future suicide. Individuals with a history of suicidal ideation are at markedly higher risk of subsequent attempts and suicide mortality, making early identification, timely intervention, and ongoing support critical components of effective suicide prevention (9). Research on suicide highlights both the complexity and heterogeneity of individuals who die by suicide. However, suicide decedents are not a homogeneous group (10). There is substantial diversity in the pathways, motivations, and circumstances leading to suicide, reflecting the interplay of psychological, social, cultural, and biological factors. Suicidal ideation is heterogeneous, encompassing different clinical presentations, levels of suicidal intent, methods, and trajectories. Several theoretical and clinical models have therefore been proposed to characterize distinct patterns of suicidal ideation. This heterogeneity is relevant when investigating biological correlates, as neurobiological alterations may vary according to the clinical and behavioral characteristics of suicidal acts (11). In the context of suicide, typologies help clarify variations in risk profiles, pathways, and presentations, which can guide more targeted prevention and treatment approaches (12). Authors identified different typologies of two main classical suicide patterns and four contemporary types (10).

- Sociological (classic type): The first systematic attempt to classify types of suicide was made by French sociologist Émile Durkheim in *Le Suicide* (1951). Durkheim proposed that suicidal behavior could be understood according to two social dimensions: integration, referring to the extent of an individual’s connection to society, and regulation, referring to the degree of social constraint. Based on these dimensions, he identified four types of suicide: egoistic, associated with low social integration; altruistic, associated with excessive integration into a collective; anomic, related to insufficient regulation and social disruption; and fatalistic, associated with excessive regulation and a perceived lack of alternatives (13).
- Psychoanalytic (classic type): Sigmund Freud proposed two different types of suicide, influenced by the psychodynamic perspectives of early psychological theories of his time: intentional (the desire to die is conscious) and semi-intentional (the suicidal intent is unconscious and may manifest as an accident) (14). Moreover, other psychoanalysts emphasized unconscious hostility turned inward, starting their research from Freud’s theories. Menninger identified three primary motivations for suicide: wish to kill (hostility or aggression directed outward, sometimes linked to anger, revenge; wish to die (a desire to escape unbearable psychological pain, hopelessness, or depression); wish to be killed (rooted in guilt, self-blame, and the need for punishment). According to Menninger, these motivations often coexist in individuals who die by suicide, making the person simultaneously the agent of aggression, the victim, and the one seeking relief (15).
- Psychological (contemporary type): Shneidman proposed a psychodynamic approach to suicide focused on the concept of cessation (the stopping of the potential for any further conscious experience). He introduced four subtypes of suicidal individuals: *psyde-seekers* (actively planning death), *psyde-initiators* (seeking control over an inevitable death), *psyde-ignorers* (who doubt that death ends consciousness), and *psyde-darers* (risking death through dangerous behaviors) (16).
- Behavioral (contemporary type): Henderson and Williams further expanded earlier typological models by proposing a six-factor framework for suicide-related behaviors, including depression, extra-punitive behavior, alienation, operant behavior, modeling, and avoidance. These factors encompass different psychological and interpersonal functions of suicidal behavior, ranging from self-directed hostility and perceived social exclusion to attempts to influence others, model suicidal behavior, or escape from distressing experiences (17).
- Relational (contemporary type): Leonard proposed three suicide typologies based on a developmental and relational framework, focusing on the individual’s level of independence and differentiation from parental figures. The three types-*dependent-dissatisfied*, *satisfied-symbiotic*, and *unaccepting* highlight different patterns of attachment, interpersonal relationships, autonomy, and responses to perceived relational loss or loss of control. Overall, this typology emphasizes the role of developmental and relational factors in suicidal behavior (18).
- Psychosocial (contemporary type): Shneidman further expanded his conceptualization of suicide by emphasizing the interaction between psychological and social factors. He proposed three types of suicide: *egotic*, primarily associated with internal psychological conflict; *dyadic*, arising from interpersonal conflicts or unmet needs within significant relationships; and *ageneratic*, characterized by alienation, loss of identity, or disconnection from others and a broader sense of belonging (16).
- Needs and motivations (contemporary type): Different methods of classifying suicide acts have been proposed, some of which relate to the fulfilment of unfulfilled needs, highlighting that suicidal behavior is multi-determined, encompassing intrapersonal conflicts, relational dynamics, and broader sociological and existential factors (10).

Large-scale analyses highlight that suicide risk is associated with multiple 96ping factors, including mental illness, substance use, financial and relationship stressors, medical problems, interpersonal violence, and criminality. Further differentiation has been observed based on method of death, healthcare utilization, and diagnostic history, underscoring the importance of tailoring interventions to specific subgroups (19). Overall, current evidence suggests that the main motivations for suicide in the general population revolve around:

- Escape from unbearable situations or emotional pain;
- Aggression or violence turned inward;
- Relational conflicts, loss, or alienation;
- Intrapsychic suffering, depression, and hopelessness;
- Socio-cultural or identity-related stressors.

These findings underscore the multifactorial nature of suicide and highlight the need for both universal and tailored prevention strategies (20). The Centre for Disease Control and Prevention reported that approximately half of individuals who died by suicide in 2004 had at least one diagnosed mental disorder, the most common being major depression (85.2% of cases), bipolar disorder (7.4%), and schizophrenia (3.3%) (21). Additionally, men are four times more likely to die by suicide, whereas women are more likely to attempt suicide (22). Epidemiological estimates of suicide are affected by systematic underreporting due to methodological and contextual limitations. Incomplete medical documentation, misclassification of deaths, difficulties in establishing suicidal intent, and the recording of some intentional overdoses or indirect self-injurious behaviors as accidental may contribute to an underestimation of actual suicide rates. These limitations highlight the complexity of accurately identifying and characterizing suicidal ideation (23).

### 1.1 Epidemiological background

In 2021, there were 47,346 suicide deaths in the European Union, corresponding to an average rate of 10.2 deaths per 100,000 population. Compared with 2011, the number of suicide deaths decreased by 13.3%, although substantial differences persisted across countries. Slovenia, Lithuania, and Hungary reported the highest standardized suicide rates, whereas Cyprus, Greece, and Italy had among the lowest rates (24). Suicide mortality was consistently higher among men, who accounted for approximately 77% of suicide deaths, while the largest proportion of deaths occurred among individuals aged 45–64 and those aged 65 years and older. Recent European data also indicate that suicide rates may be increasing in specific demographic groups, including women under 20 and men over 85 (25). In Italy, epidemiological data for 2020–2021 indicate 7,422 suicide deaths. Although Italy generally reports lower suicide rates than several other European countries, substantial regional and demographic differences persist (26). Men accounted for approximately 77% of suicide deaths, whereas women showed higher rates of non-fatal suicide attempts (27). Regional differences have also been reported, with higher suicide rates in some northern regions compared with southern areas, suggesting that socioeconomic, cultural, healthcare, and social-support factors may contribute to the observed variability (28).

### 1.2 Method choice and intent

Globally, between 15% and 20% of suicides involve pesticide self-poisoning, particularly in countries where highly hazardous pesticides are widely available. Restricting access to highly hazardous pesticides has therefore been identified as an important and cost-effective suicide prevention strategy (4). Sex differences in suicide mortality have been partly attributed to the differential use of methods. Females are more likely to use methods with lower lethality, whereas males more frequently employ violent and highly lethal methods, contributing to the observed difference in suicide mortality between the sexes (29). Evidence reported by Spicer et al. indicates that suicide methods differ markedly in lethality, with firearms, drowning, and hanging/asphyxiation showing the highest case-fatality rates, while drug overdose or poisoning and cutting or piercing are associated with comparatively lower lethality (30). A systematic review published in 2022 demonstrated pronounced differences in case-fatality rates across suicide methods, with firearms showing the highest lethality (89.7%), followed by hanging/asphyxiation and drowning. Intermediate lethality was observed for gas poisoning and jumping from height, whereas substantially lower case-fatality rates were associated with drug or liquid poisoning and cutting (31). Methods that are potentially interruptible or reversible, including poisoning and cutting, generally exhibit lower lethality, whereas those leading to rapid physiological collapse, such as firearms or jumping from height, are associated with significantly higher fatality rates. This distinction underscores the critical role of intervention windows in determining outcomes (32). Suicide methods are influenced by factors such as the speed and severity of physiological effects and the possibility of timely intervention. Data from 16 European countries similarly showed differences in method selection between men and women: hanging was the most frequently used method, while intentional poisoning was more common among women. Firearms were substantially more frequently used by men and were associated with high lethality (33). Overall, these findings highlight the importance of considering both the method and lethality of suicidal ideation when characterizing suicide attempts and their clinical and biological correlates.

### 1.3 Suicide attempts

Estimates from the WHO indicate that, for each death by suicide, more than 20 suicide attempts occur, highlighting the substantial burden of non-fatal suicidal ideation and highlighting the substantial burden of non-fatal suicidal behavior and its strong predictive value for future suicidality (34). Suicidal thoughts and behaviors (STB) encompass a broad range of phenomena, from passive or active suicidal ideation to self-directed actions carried out with some intent to die. Although suicidal ideation and behavior have traditionally been conceptualized along a continuum, the progression from ideation to suicidal behavior is not necessarily linear (35). For each death by suicide, there are substantially more non-fatal suicide attempts. Among the various risk factors identified, a history of previous suicide attempts is consistently recognized as one of the most robust predictors of suicide in the general population (2). A substantial proportion of individuals who engage in a first suicide attempt, exceeding one-third, subsequently repeat the behavior, with approximately 1 in 15 eventually dying by suicide, underscoring the strong predictive value of prior attempts for future suicide risk (36). More than 90% of suicide attempts are linked to the presence of psychiatric conditions, most commonly major depressive disorder, bipolar disorder, anxiety disorders, personality disorders, schizophrenia and other psychotic disorders, and substance use disorders, underscoring the central role of mental illness in suicidal ideation (37–39). Individuals with multiple suicide attempts history represent a clinically distinct subgroup compared with single attempters, characterized by greater psychopathological burden and a more complex risk profile. In particular, a higher lethality of suicidal acts has been observed among multiple attempters, suggesting a potential escalation in the severity of suicidal ideation over time (40). Suicidal behavior is also strongly associated with psychiatric conditions, particularly mood, anxiety, personality, psychotic, and substance use disorders These findings reinforce the contribution of mood disorder severity, particularly within bipolar and depressive spectrum conditions, as a critical component in the assessment of suicide risk beyond the mere presence of psychiatric diagnosis (41). Meta-analytic evidence indicates that most traditional risk factors for suicidality, particularly depressive symptoms, hopelessness, and sociodemographic variables, are more effective in distinguishing individuals with suicidal ideation from non-suicidal populations than in differentiating those who progress to suicide attempts. In contrast, only a limited number of factors, including anxiety disorders (notably PTSD), substance use, and history of sexual trauma, show modest associations with the transition from ideation to action. These findings support an “*ideation-to-action*” framework, suggesting that the progression to suicide attempts is not merely a function of symptom severity but likely involves additional mechanisms, such as increased capability for self-harm or exposure to painful and provocative experiences (42).

### 1.4 Suicide in children, teens and adolescents

Suicide represents a major public health concern and remains one of the leading causes of death among young people. In 2021, it was the third leading cause of death globally among individuals aged 15–29 years. In the European Union, the highest crude suicide rates among young people were observed in the 25-29-year age group, followed by those aged 20–24 and 15–19 years (2). Suicide risk varies considerably across the lifespan, with age and sex playing important roles. Global data among individuals aged 10–24 years indicate substantial geographic disparities in suicide rates and trends, with males generally showing higher rates than females (43). Suicide is the second leading cause of death among individuals aged 15–24 years, with most affected youths presenting with underlying psychiatric disorders, particularly depression. In children, suicidal behaviors are often impulsive and linked to emotional dysregulation, whereas in adolescents they are more commonly associated with psychosocial stressors such as interpersonal conflict, academic pressure, and perceived failure. Key risk factors include family history of suicide, exposure to violence, impulsivity, bullying, access to firearms, and feelings of hopelessness, with acute stressors often acting as triggers. However, suicidal ideation, including ideation and attempts, may show different patterns, with some studies reporting higher prevalence among female and older adolescents. According to data from the Global Student Health Survey, the one-year prevalence of suicidal ideation and suicide attempts among adolescents was 16.9% and 17%, respectively. Furthermore, the data indicate that older adolescents and female adolescents exhibited a higher prevalence of suicidal behaviors (44). Suicidal ideation during adolescence is multifactorial and reflects the interaction of individual, interpersonal, psychiatric, and environmental determinants. Psychiatric disorders, particularly depressive and other affective disorders, are important correlates, while anxiety, substance use, family dysfunction, bullying, social isolation, impulsivity, acute stressors, and exposure to violence may also contribute to risk (45). These factors may vary across developmental stages and socioeconomic contexts, with adolescents in low- and middle-income countries experiencing a disproportionate burden of suicidal ideation (1). Notably, a substantial proportion of adolescent suicide attempts may occur without preceding suicidal ideation, further illustrating the complexity and heterogeneity of suicidal behavior (46).

### 1.5 Suicide in young adults/adults

Suicide in adulthood remains a significant public health challenge, with a complex interplay of clinical, psychosocial, and sociodemographic determinants. Systematic evidence from psychological autopsy studies demonstrates that the presence of any mental disorder is the strongest individual-level predictor of suicide mortality in adults, conferring more than a tenfold increase in risk compared with those without psychiatric diagnoses; a history of self-harm similarly confers a markedly elevated risk. Other factors, including adverse life events, family history, and socioeconomic circumstances, show additional associations with suicidal ideation, underscoring its multifactorial nature (47). Large-scale population-based data indicate that suicidal thoughts and behaviors in adults exhibit substantial variability in suicidal thoughts and behaviors across sociodemographic groups. Suicidal behaviors demonstrate clear sociodemographic gradients. Higher prevalence is consistently observed among females for suicidal ideation, planning, and attempts, while younger adults (particularly those aged 18–39 years) show higher rates compared with older age groups. Differences have also been reported according to racial and ethnic background, socioeconomic circumstances, geographic location, and access to healthcare. Overall, these findings highlight the importance of considering both individual clinical characteristics and broader contextual factors when investigating suicidal ideation in adults (48).

### 1.6 Suicide in older adults

Suicide rates generally increase with age and often peak in the oldest age groups, particularly among males. Across Europe, the male-to-female suicide rate ratio typically ranges from 3:1 to 7:1, although this gender gap varies across regions. The oldest age group (85+) has consistently exhibited the highest suicide rates, particularly among males, underscoring their increased vulnerability (43, 49).

Clinically severe suicide attempts, which carry a substantial risk of death, are more common among older adults than in younger populations. This trend likely reflects a higher degree of planning and reduced ambivalence regarding death among the elderly. Bereavement, chronic illness, and disability likely contribute to the elevated suicide risk in this age group. Older individuals who attempt suicide frequently exhibit cognitive deficits, particularly impaired cognitive control. For instance, a research group investigated cognitive inhibition in elderly individuals who had engaged in high-lethality suicide attempts and who closely resembled suicide victims in behavioral characteristics. The findings suggested that diminished cognitive control in high-lethality attempters may undermine their ability to effectively cope with real-life problems, leading to a cumulative and potentially catastrophic build-up of stressors (50). Clinical studies further indicate that individuals with a history of suicidal ideation tend to exhibit greater depressive symptom severity and higher levels of suicidal intent than psychiatric controls with similar diagnoses. These findings highlight the relevance of affective symptom severity and acute clinical state in characterizing suicide risk, particularly among individuals with mood disorders (51). In older populations, multifaceted interventions combining clinical management, psychological approaches, and social support have shown potential for reducing suicidal ideation and behavior, although findings remain heterogeneous (52).

### 1.7 Neurobiology in suicide

A substantial proportion of suicides occur impulsively during periods of acute crisis, reflecting a transient impairment in the capacity to manage psychosocial stressors, including financial strain, relational conflicts, and chronic pain or medical illness. Robust evidence, particularly from high- income countries, has consistently demonstrated a strong association between suicide and psychiatric disorders, most notably depressive disorders and alcohol use disorders, as well as a history of prior suicide attempts (2). Suicidal ideation has been linked to dysfunction across multiple neurotransmitter systems, particularly the dopaminergic, serotonergic, and noradrenergic pathways. Alterations in these systems are commonly investigated by measuring the concentrations of neurotransmitters and their metabolites in biological fluids, including cerebrospinal fluid (CSF), blood, and urine. Among these, CSF measurements are generally considered the most reliable indicators of central neurotransmitter activity. Cumulative evidence from reviews and meta-analyses has consistently demonstrated abnormalities in the CSF concentrations of key monoamine metabolites in individuals exhibiting suicidal ideation. These include homovanillic acid (HVA), a dopamine metabolite, 5-hydroxyindoleacetic acid (5-HIAA), a serotonin metabolite and 3-methoxy-4- hydroxyphenylglycol (MHPG), a metabolite of norepinephrine (53). Altered levels of these metabolites have been significantly associated with suicide in patients with depressive disorders, whereas more limited and selective abnormalities have been observed in other psychiatric conditions, such as substance use disorders (primarily involving 5-HIAA and HVA) and schizophrenia (mainly involving 5-HIAA). Notably, reduced CSF levels of 5-HIAA have been associated with an approximately 4.5-fold increased risk of death by suicide, further supporting the role of serotonergic dysfunction as a key neurobiological correlate of suicidal ideation (54). Levels of 5-HIAA, HVA, and MHPG were all inversely correlated with the maximum lethality of suicide attempts, suggesting that deficits in serotonergic, dopaminergic, and noradrenergic neurotransmission may contribute to the severity of suicidal ideation (38). Abnormalities in serotonergic neurotransmission have been consistently associated with impaired impulse control, heightened aggression, and an increased risk of suicidal behavior, highlighting the central role of serotonin in the neurobiological underpinnings of suicide. Sher, in 2022, reported that lower CSF levels of 5-HIAA have been reported in individuals who engaged in high-lethality and violent suicide attempts compared with those with low-lethality attempts. Reduced serotonergic activity was associated with more lethal suicidal ideation (55). CSF levels of 5-HIAA are widely regarded as an indirect marker of central serotonin function. Differences in 5-HIAA concentrations may be attributable to variability in serotonergic innervation and to changes in serotonin turnover and metabolic processes. Notably, both genetic predisposition and environmental influences contribute to the regulation of CSF 5-HIAA levels (56). The link between suicidality and serotonergic dysfunction has been widely interpreted in the context of impaired aggression regulation. Specifically, the serotonin hypothesis posits that disinhibited aggression results from a functional imbalance between diminished prefrontal inhibitory control and increased limbic system responsiveness to emotional stimuli, thereby contributing to suicidal ideation (57). Notably, sex-dependent differences have been reported, with CSF 5-HIAA levels inversely associated with childhood exposure to violence in females but not in males. Early-life stress exerts a significant impact on the serotonergic system, a key neurobiological substrate underlying behavioral inhibition, impulsivity, and aggression. Evidence from both preclinical and clinical studies indicates that childhood adversity, whether physical or psychological, is linked to enduring dysregulation of serotonergic pathways (58). Data reported by another study published by Sher et al., also examined the relationship between CSF levels of 5-HIAA, HVA, and MHPG and the maximum lethality of suicide attempts in patients with bipolar disorder (55). Recent meta-analytic evidence supports the involvement of central serotonergic and dopaminergic dysfunction in suicide risk, as indexed by cerebrospinal fluid monoamine metabolites. A comprehensive systematic review and random-effects meta-analysis encompassing nearly 2,000 individuals who attempted suicide and over 1,200 psychiatric controls found that CSF concentrations of both 5- HIAA, the principal serotonin metabolite, and homovanillic acid, a major dopamine metabolite, were significantly lower in individuals who had attempted suicide compared with non-attempters (59). Specifically, standardized mean differences indicated moderate reductions in both 5-HIAA and HVA among attempters, suggesting that deficits in serotonergic and dopaminergic turnover may contribute to the biological substrate of suicidal ideation. Importantly, the association was more robust and consistent for 5-HIAA, whereas findings for HVA were more heterogeneous across studies. These alterations appear to be particularly pronounced in individuals engaging in violent or high-lethality attempts, where lower CSF 5-HIAA has been observed relative to non-violent attempters, implying a link between reduced serotonergic activity and behavioral disinhibition, impulsivity, and the severity of suicidal acts. Such findings align with broader neurobiological models in which impaired serotonin turnover diminishes inhibitory control over affective and aggressive impulses, thereby elevating suicide risk. Although HVA reductions have been less consistent, dopaminergic dysregulation may influence motivational and reward-related processes relevant to suicidal decision-making (60). Overall, while methodological limitations such as small sample sizes and heterogeneity in psychiatric diagnoses persist, these meta-analytic data underscore the relevance of CSF 5-HIAA, and to a lesser extent HVA, as biomarkers reflecting monoaminergic contributions to suicide risk. The evidence supports a model in which concurrent serotonergic and dopaminergic abnormalities contribute to neurochemical vulnerability for suicidal behavior, with serotonergic deficits showing the strongest and most reproducible association in clinical samples (59).

## 2 Materials and methods

The literature search was conducted using Medline/PubMed and Google Scholar. Searches were performed between February 2026 and April 2026 and covered publications from January 2005 to April 2026. The publication period from January 2005 to April 2026 was selected to focus the primary evidence synthesis on contemporary studies and recent methodological and clinical developments in the investigation of biological correlates of suicidal ideation. Earlier seminal studies were considered as historical background where relevant to contextualize the development of the field, but they were not eligible for inclusion in the primary evidence synthesis. Accordingly, studies published before 2005 were not included among the studies contributing to the qualitative synthesis unless explicitly cited for historical or contextual purposes. The exact search dates for each database were recorded and are reported below. For Medline/PubMed, the following Boolean search string was applied to title and abstract fields:

(Suicide[tiab] OR “suicidal behavior”[tiab] OR “suicidal behavior”[tiab] OR “self-harm”[tiab] OR depression[tiab] OR “suicide attempt”[tiab] OR lethality[tiab] OR “CSF biomarkers”[tiab]) AND (“5-HIAA”[tiab] OR “5-hydroxyindoleacetic acid”[tiab] OR HVA [tiab] OR “homovanillic acid”[tiab] OR “neurobiological alterations”[tiab])

The PubMed search (on February 2026 and April 2026) used free-text terms restricted to the title and abstract fields; no controlled vocabulary, including Medical Subject Headings (MeSH) terms, was applied. The PubMed search identified 66 articles. For Google Scholar, searches were conducted in March 2026, given the platform’s limited support for complex Boolean syntax, searches were conducted using combinations of free-text phrases reflecting the same conceptual domains as the PubMed strategy, including (e.g., “suicide AND 5-HIAA”, “suicidal behavior AND HVA”, “suicide AND CSF biomarkers”). Google Scholar identified 48 articles. Because Google Scholar provides more limited support for complex Boolean search strategies, individual combinations of relevant terms were searched separately. The number of records retrieved from each database was recorded separately. Duplicate records identified across the two databases were removed before screening. After deduplication using Microsoft Excel Software, the authors removed 34 articles and 80 unique records remained for screening. Study selection was conducted in two stages. First, titles and abstracts were screened independently by two reviewers (LS and RT) to identify records potentially relevant to the review question. Potentially eligible studies were subsequently assessed by the same reviewers through full-text review according to the predefined inclusion and exclusion criteria. Disagreements between the two reviewers were discussed and resolved by consensus; when consensus could not be reached, a third reviewer was consulted (LTM). A total of 19 records were excluded at the title/abstract stage. The remaining 61 records were assessed in full text, of which 38 were excluded. The main reasons for full-text exclusion were: (i) biomarker concentrations were reported without a direct relationship to suicidal ideation, suicide attempts, lethality, or another predefined suicide-related outcome; (ii) suicide was addressed only as a general clinical feature, without examination of a relevant biological or biomarker correlate; and (iii) the study did not otherwise meet the predefined eligibility criteria. The remaining 23 studies were included in the qualitative synthesis (**Table 1**; **Figure 1**).

**Table 1:** Characteristics and main findings of the studies included in the narrative review.

	Title	Authors	Research purpose	DOI/Ref	Year	Findings
1	Associated factors of depression, anxiety, and suicide behavior among men in Switzerland: findings from the Swiss health survey 2022	Laskowski NM, Müller R, Theunert M and Paslakis G	To investigate whether the demographic, socioeconomic, and psychosocial correlates of depression, anxiety, and suicide attempts among men in Switzerland, using nationally representative data from the 2022 Swiss Health Survey.	10.3389/fpsyg.2026.1 725181	2026	This analysis reported that lifetime suicide attempts among adult men were relatively rare but were associated with specific sociodemographic and psychosocial factors. Higher risk was observed in gender-diverse and sexual minority individuals, as well as in those with lower education, unemployment, lack of a stable relationship, and migration background. Anxiety and depression symptoms were more frequent among individuals with the same vulnerability profiles. Protective factors included higher education and being in a relationship, both associated with lower distress and reduced suicide risk. Overall, suicidal ideation appeared to cluster within specific high-risk subgroups characterized by combined psychological and social vulnerabilities (61)
2	Degree of Suicide Intent and the Lethality of Means Employed: A Study of Chinese Attempters	Zhang J, Xu H	This study assessed whether the level of suicidal intent is associated with the lethality of the methods used in suicide attempts.	10.1080/13811110701 541889	2007	This study found that drug ingestion was the predominant method used in suicide attempts, followed by cutting, pesticide ingestion, gas inhalation, and jumping from height. Overall, methods of low-to-moderate lethality were most common, reflecting availability in the studied setting. A clear gradient in lethality was observed, with jumping and gas inhalation representing the most lethal methods, and cutting and drug ingestion the least. Suicidal intent was positively associated with method lethality, indicating that individuals with higher intent tended to select more lethal means. No significant gender differences were observed in method selection. Psychiatric morbidity and method lethality were significant predictors of suicidal intent, while marital status was not. Interpersonal conflicts were the most frequently reported precipitating factors, whereas economic hardship was associated with the highest levels of suicidal intent. Overall, findings indicate that method choice is influenced by intent, availability, and contextual factors, with lethality of the method playing a key role in the severity of suicide attempts (62)
3	Adolescent health lifestyles and suicidality: Emerging risks in a latent class analysis	Bitar S, G. Mendes F, Lopes Ferreira J, Samuel R, Catunda C	This study identified distinct adolescent health lifestyle patterns and examined their associations with suicidal ideation and suicide attempts using data from the 2022 Luxembourg Health Behavior in School-aged Children survey. Latent class analysis was used to derive five lifestyle profiles based on multiple health-related behaviors, and hierarchical logistic regression was applied to evaluate their relationships with past-year suicidal ideation and suicide attempts.	0.1017/gmh.2026.10147	2026	This study identified five distinct adolescent lifestyle profiles based on dietary habits, physical activity, substance use, and digital engagement. Profiles characterized by health-promoting behaviors showed low levels of substance use and problematic digital behavior, whereas risk profiles included polysubstance use, combinations of e-cigarette use with problematic social media engagement, high alcohol consumption, and low adherence to healthy lifestyle practices. Suicidal ideation and suicide attempts were more prevalent among adolescents with clustered risk behaviors, particularly those involving polysubstance use or the combination of digital vulnerabilities with unhealthy lifestyles. Regression analyses confirmed that engagement in multiple substance use behaviors and problematic digital behaviors were associated with higher odds of both suicidal ideation and attempts. In contrast, alcohol use alone was mainly associated with suicidal ideation without a clear increase in suicide attempts, and adolescents with no substance use did not show elevated risk. Overall, suicidal ideations were most strongly associated with profiles characterized by the co-occurrence of multiple behavioral risk factors rather than isolated lifestyle behaviors (63)
4	Life event trajectories and suicidal ideation throughout adolescence: a prospective cohort study from Germany, France, Ireland, and the UK	Chen, Pei-Jung et al.	This study investigated the relationship between cumulative and domain-specific patterns of life events during adolescence and suicidal ideation.	10.1016/S2215-0366(25)00359-1	2026	This longitudinal study identified four trajectories of cumulative life event exposure during adolescence (high, mid-high, mid-low, and low) and demonstrated that persistent high exposure was associated with the greatest risk of suicidal ideation over time. Adolescents in the high-exposure trajectory more frequently came from socioeconomically disadvantaged backgrounds and reported higher rates of childhood maltreatment, reflecting a clustering of environmental vulnerabilities. Suicidal ideation was consistently highest and stable across time in this group, while intermediate and low exposure groups showed lower and comparable levels of risk. Trajectory membership remained a significant predictor of suicidal ideation even after adjustment for sociodemographic and adverse childhood factors, and model performance improved when cumulative life event exposure was included. The association appeared independent of childhood maltreatment and stable across developmental stages. Family-related stressors emerged as particularly influential in predicting suicidal ideation. Overall, findings indicate that a persistently high cumulative burden of life events represents a key determinant of suicidal ideation, suggesting a possible threshold effect whereby elevated and sustained stress exposure substantially increases vulnerability (64)
5	Mapping the genetic landscape of suicide risk: Insights from genomic SEM	Yao K, Zhuo C, Chen X, Li C, Song H, Zou J, Tian H	The study examined and characterized the genetic architecture underlying suicide-related risk by identifying causal genetic variants and genes associated with a latent “high-risk suicide syndrome” phenotype. To achieve this, the researchers integrated genomic structural equation modeling with post-GWAS analytical approaches to detect independent, genome-wide significant loci beyond previously known associations. They further sought to fine-map susceptibility genes using transcriptome-wide association analyses at tissue and cellular levels, investigate regulatory mechanisms influencing gene expression, and evaluate genetic pleiotropy through correlations with a wide range of common diseases. Additionally, the study aimed to quantify the contribution of genetic variation using polygenic risk scores and to provide a comprehensive description of the heritable and polygenic components contributing to suicide risk.	10.1016/j.neurot. 2026.e00 852	2026	This genomic analysis demonstrated that multiple suicide-related traits share a common underlying genetic liability, supporting a transdiagnostic model of suicidality. A single latent factor encompassing suicidal behavior, depression, childhood trauma, loneliness, financial hardship, suicidal ideation, and chronic pain showed strong model fit, indicating substantial shared genetic architecture across these phenotypes. Genome-wide analyses identified a robust polygenic signal with numerous associated loci and genes, confirming the highly polygenic nature of suicide risk. Fine-mapping and transcriptome-wide analyses highlighted several candidate variants and genes with potential causal roles, including those involved in protein ubiquitination, DNA repair, synaptic function, intracellular trafficking, and cellular stress regulation. Overall, findings suggest that genetic susceptibility to suicidal behavior is distributed across multiple biological pathways rather than being driven by a single mechanism, reflecting the convergence of systems involved in neuronal integrity, synaptic activity, and cellular homeostasis (65)
6	Cross-national associations between gender and mental disorders in the World Health Organization World Mental Health Surveys	Seedat S, Scott KM, Angermeyer MC, Berglund P, Bromet EJ, Brugha TS, Demyttenaere K, de Girolamo G, Haro JM, Jin R, Karam EG, Kovess-Masfety V, Levinson D, Medina Mora ME, Ono Y, Ormel J, Pennell BE, Posada-Villa J, Sampson NA, Williams D, Kessler RC.	The study aimed to examine how gender differences in the lifetime prevalence of DSM-IV mental disorders vary across birth cohorts and countries, and to assess whether this variability is associated with differences in gender role traditionality, as reflected by indicators such as female education, employment, age at marriage, and contraceptive use.	10.1001/archgenpsychiatr y.2009.36	2009	This large multinational survey analysis demonstrated consistent gender differences in the prevalence of mental disorders across countries and over time. Women showed a higher lifetime risk of internalizing disorders, particularly mood and anxiety disorders, whereas men exhibited higher prevalence of externalizing and substance-related disorders. No significant gender differences were observed for bipolar disorder. These patterns were broadly consistent across countries, although the magnitude of differences varied. Cohort analyses indicated that gender disparities were generally stable over time for most disorders; however, the female excess in major depressive disorder and the male predominance in substance use disorders showed a modest narrowing in more recent cohorts. Further analyses incorporating a composite measure of gender role traditionality indicated that shifts toward less traditional gender roles were associated with reduced gender differences in these conditions. Specifically, decreasing traditionality corresponded to a convergence in male and female risks for both depression and substance-related disorders, with temporal trends playing a key role for depression and cross-country variation contributing more to substance use patterns. Overall, findings suggest that while gender differences in mental disorders remain robust, they may be influenced by sociocultural changes over time, particularly those related to evolving gender roles (66)
7	CSF monoamine metabolites and lethality of suicide attempts in depressed patients with alcohol dependence	Sher L, Oquendo MA, Grunebaum MF, Burke AK, Huang YY, Mann JJ	The study identified the relationship between CSF monoamine metabolites and the lethality of suicide attempts in depressed individuals with comorbid alcoholism by measuring CSF concentrations of monoaminergic markers and comparing them across groups stratified by attempt lethality.	10.1016/j.euroneuro.2006.04.011	2007	High- vs. low-lethality individuals who attempted suicide did not differ on demographic or most clinical variables, except for a higher number of prior psychiatric hospitalizations in the high-lethality group. CSF analysis showed significantly lower 5-HIAA levels in high-lethality attempters (≈23.5% reduction), remaining significant after adjustment for age and sex, while no differences were observed for HVA or MHPG. CSF monoamine metabolites were not correlated with depression severity or suicidal ideation. Overall, reduced CSF 5-HIAA was specifically associated with greater lethality of suicide attempts (38)
8	A prospective study of the association of cerebrospinal fluid monoamine metabolite levels with lethality of suicide attempts in patients with bipolar disorder	Leo Sher, Juan J Carballo, Michael F Grunebaum, Ainsley K Burke, Gil Zalsman, Yung-yu Huang, J John Mann, Maria A Oquendo	The study assessed bipolar patients by measuring CSF monoamine metabolites (5-HIAA, HVA, and MHPG) at baseline and examining their relationship with the maximum lethality of suicide attempts over a 2-year follow-up period.	10.1111/j.1399-5618.2006.00319.x	2006	In this prospective follow-up of bipolar depressed patients, baseline comparisons showed no differences between those who later attempted suicide and those who did not in demographic or depressive symptom variables, although future attempters exhibited higher aggression, hostility, and familial history of suicidal ideation. Baseline CSF levels of 5-HIAA, HVA, and MHPG did not differ between groups. However, correlational analyses demonstrated that lower baseline CSF concentrations of 5-HIAA, HVA, and MHPG were significantly associated with greater lethality of suicide attempts during follow-up. No associations were found between CSF metabolites and baseline clinical measures. Overall, reduced monoaminergic activity was linked to the severity of suicidal ideation rather than its occurrence (38)
9	Disentangling the Effects of Suicide Attempts and Psychiatric Diagnosis Based on a Genotype-Informed Dynamic Model of the Serotonin Presynapse	Radenković L, Pantović-Stefanović M, Brajušković G, Ivković M, Savić-Pavićević D, Pešović J.	The aim of the study was to disentangle the respective contributions of suicide attempt history and psychiatric diagnosis on serotonergic presynaptic dynamics using a genotype-informed computational model of the 5-HT system.	10.3390/genes16101141	2025	No significant associations were found between modelled serotonergic features and suicide attempt history when analyzed across the entire sample. In contrast, psychiatric diagnosis significantly influenced multiple serotonergic parameters, particularly those related to 5-HIAA dynamics. Differences in serotonergic profiles were observed between diagnostic groups, indicating that serotonin metabolism varies primarily according to underlying psychiatric condition. Interaction effects between diagnosis and suicide attempt status were identified for selected features, suggesting diagnosis-dependent relationships between serotonergic dynamics and suicidality (67)
10	Tryptophan and Kynurenine Metabolites: Are They Related to Depression?	Pompili M, Lionetto L, Curto M, Forte A, Erbuto D, Montebovi F, Seretti ME, Berardelli I, Serafini G, Innamorati M, Amore M, Baldessarini RJ, Girardi P, Simmaco M.	To aim of the authors was to examine whether previously reported alterations in tryptophan–kynurenine metabolism (including decreased tryptophan and increased kynurenine and related metabolites), possibly linked to inflammatory processes, are present in major depressive disorder and whether such changes may be associated with suicidal behavior.	10.1159/000491604	2019	Pompili et al. found that patients with major affective disorders showed significantly higher plasma tryptophan and lower kynurenine levels compared with healthy controls, resulting in an increased TRY/KYN ratio. Other kynurenine pathway metabolites, including 5-HIAA, did not differ significantly between groups, although the 5-HIAA/tryptophan ratio was reduced in depressed patients. No differences in metabolite levels were observed according to history of suicide attempts, indicating no association between these biochemical markers and suicidal behavior. Overall, the study suggests altered tryptophan–kynurenine metabolism in depression without clear evidence of reduced serotonergic activity or links to suicidality (68)
11	A role for inflammatory metabolites as modulators of the glutamate N-methyl-D-aspartate receptor in depression and suicidality	Bay-Richter C, Linderholm KR, Lim CK, Samuelsson M, Träskman-Bendz L, Guillemin GJ, Erhardt S, Brundin L.	The authors tested whether depressive symptoms and suicidality fluctuate over time in individuals prone to suicidal behavior, in relation to cerebrospinal fluid (CSF) levels of inflammatory markers and kynurenine pathway metabolites.	10.1016/j.bbi.2014.07.012	2014	Individuals with recent suicide attempts exhibited persistent dysregulation of the kynurenine pathway, characterized by significantly increased CSF quinolinic acid (QUIN) and decreased kynurenic acid (KYNA) levels compared with controls, with these alterations remaining over time. KYNA levels were inversely associated with depressive symptom severity and suicidal ideation, whereas QUIN showed no consistent correlation with clinical measures. Interleukin-6 (IL-6) levels were positively associated with suicidal symptom severity. Overall, findings indicate a sustained imbalance toward increased neurotoxicity and reduced neuroprotection, with immune–metabolic abnormalities linked to suicidality (69)
12	Risk factors for suicide among patients with schizophrenia: a cohort study focused on cerebrospinal fluid levels of homovanillic acid and 5-hydroxyindoleace tic acid	Neider D, Lindström LH, Bodén R.	The objective of the study was to investigate the association between CSF monoamine metabolites (5-HIAA and HVA), exposure to bullying, and subsequent suicide in patients with schizophrenia.	10.2147/NDT.S107178	2016	During long-term follow-up, 12% of patients died by suicide. No significant differences were observed in absolute CSF levels of HVA or 5-HIAA between suicide and non-suicide groups. However, the HVA/5-HIAA ratio was significantly lower in individuals who died by suicide, indicating a relative imbalance between dopaminergic and serotonergic activity. Exposure to childhood bullying was significantly more frequent among those who died by suicide, while no associations were found with other sociodemographic or clinical variables. Overall, both neurochemical imbalance and early-life adversity were associated with increased long-term suicide risk (70)
13	CSF Apolipoprotein E in attempted suicide	Sellus P, Nordström P, Nordström AL, Jokinen J.	The aim of the authors was to investigate whether cerebrospinal fluid apolipoprotein E (ApoE) levels are associated with the severity of suicidal behavior, including number of previous suicide attempts, reversibility/interruption of attempts, and use of violent methods.	10.1016/j.jad.2017.08.019 .	2018	The authors reported that CSF ApoE levels in individuals who attempted suicide were not associated with sex, depression severity, or history of previous attempts. However, lower CSF ApoE concentrations were significantly correlated with greater irreversibility of suicide attempts, indicating an association with more lethal methods. A trend toward lower ApoE levels was also observed in individuals using violent methods, while no significant relationships were found with attempt repetition or interruption. Importantly, CSF ApoE levels were not correlated with CSF 5-HIAA, suggesting that the observed associations with lethality are independent of serotonergic activity. Overall, lower CSF ApoE appears linked to increased lethality of suicidal ideation without involvement of 5-HIAA (71)
14	Elevated serotonin and 5-HIAA in the brainstem and lower serotonin turnover in the prefrontal cortex of suicides	Bach H, Huang YY, Underwood MD, Dwork AJ, Mann JJ, Arango V.	The aim of the authors was to compare brainstem serotonin (5-HT) and its metabolite 5-HIAA levels between suicides and nonpsychiatric sudden-death controls, in order to assess regional serotonergic differences across the dorsal and median raphe nuclei.	10.1002/syn.21695	2014	The authors found region-specific differences in serotonergic markers in postmortem brain tissue from suicides compared with controls. In the brainstem, suicides exhibited higher levels of serotonin and 5-HIAA, but a significantly reduced 5-HIAA/5-HT ratio, indicating decreased serotonergic turnover. These differences were independent of postmortem variables. In the prefrontal cortex, no significant differences in serotonin or 5-HIAA were observed, although a non-significant reduction in 5-HIAA and turnover ratio was noted. Additionally, an inverse correlation between brainstem and cortical serotonergic measures was observed. Overall, the findings suggest altered regional serotonergic regulation in suicide rather than a global reduction in 5-HIAA (72)
15	CSF 5-HIAA, cortisol and DHEAS levels in suicide attempters	Chatzittofis A, Nordström P, Hellström C, Arver S, Åsberg M, Jokinen J.	The authors aimed to examine CSF 5-HIAA together with CSF and plasma cortisol and DHEAS in medication-free individuals who attempted suicide compared with healthy controls, and to assess their associations with childhood trauma and subsequent suicide outcomes in a longitudinal framework. The study evaluated whether neuroendocrine markers differed between groups and whether baseline levels of CSF cortisol, 5-HIAA, or DHEAS were associated with later suicide mortality, alongside analyses of the relationship between these biological measures and exposure to childhood adversity.	10.1016/j.euroneuro.2013 .02.002	2013	Individuals who attempted suicide showed higher CSF and plasma cortisol levels compared with controls, indicating HPA axis hyperactivity, while CSF DHEAS did not differ overall except for higher levels in males. CSF 5-HIAA levels were reduced in attempters, with significant differences observed in females. Correlations indicated relationships among cortisol, DHEAS, and a trend with 5-HIAA. Childhood violence exposure was associated with altered stress markers, including a lower cortisol/DHEAS ratio. Follow-up suggested that individuals who completed suicide tended to have lower CSF 5-HIAA and higher cortisol levels, supporting a link between serotonergic and HPA axis dysregulation and suicidal outcomes (73)
16	CSF 5-HIAA and exposure to and expression of interpersonal violence in suicide attempters	Moberg T, Nordström P, Forslund K, Kristiansson M, Asberg M, Jokinen J.	The authors aimed to examine the relationship between CSF 5-HIAA levels and exposure to interpersonal violence, as well as the expression of violent behavior during childhood and adulthood, assessed using the Karolinska Interpersonal Violence Scale (KIVS). The study evaluated how CSF serotonergic activity was associated with violence-related experiences and behaviors across the lifespan, including analyses of associations with childhood trauma and adult violent expression.	10.1016/j.jad.2011.01.018 .	2011	In medication-free individuals who attempted suicide, CSF 5-HIAA levels measured via GC-MS did not vary by sex, age, or height. Childhood exposure to violence was associated with higher rates of Axis II personality disorders and greater depression severity. A sex-specific association was observed between childhood trauma and CSF 5-HIAA, with a significant negative correlation in women but not in men. When stratified by trauma exposure, higher childhood violence was linked to lower CSF 5-HIAA, particularly among traumatized individuals. Lower CSF 5-HIAA was also associated with greater expressed violent behavior in adulthood in those with low metabolite levels, while no significant associations were found in the overall sample after adjustment for covariates (74)
17	CSF biomarkers in suicide attempters-a principal component analysis	Lindqvist D, Janelidze S, Erhardt S, Träskman-Bendz L, Engström G, Brundin L.	The aim of the authors was to identify underlying biological patterns among a panel of 20 cerebrospinal fluid (CSF) biomarkers in individuals who attempted suicide and to examine their associations with suicidal behavior.	10.1111/j.1600-0447.2010.01655.x.	2010	Four independent biomarker-derived factors were identified. A composite factor characterized by high CSF levels of monoamine metabolites (5-HIAA and HVA) and IL-6, and low orexin (Factor 4), was significantly associated with violent suicide attempts and an increased risk of deaths by suicide during follow-up; higher scores on this factor were also linked to lower impulsivity. Individuals in the highest tertile of this factor showed a greater incidence of suicide. A separate factor reflecting pro-inflammatory cytokines (Factor 2) was positively correlated with suicidal intent (SIS scores). Elevated scores on immune-related factors were associated with greater depressive symptom severity. No significant differences in biomarker factor scores were observed across diagnostic categories, and most factors were not associated with overall symptom severity except in severe depression. Overall, specific neurobiological profiles involving monoaminergic, inflammatory, and neuropeptide systems were associated with dimensions of suicidal ideation and long-term risk (75)
18	Early death and CSF monoamine metabolites in schizophrenia spectrum psychosis	Carlborg A, Jokinen J, Nordström AL, Jönsson EG, Nordström P.	The authors investigated whether cerebrospinal fluid (CSF) concentrations of 5-HIAA and homovanillic acid (HVA) are associated with early mortality.	10.3109/08039488.2010.5 03903	2011	The authors showed that, over long-term follow-up in patients with schizophrenia spectrum psychosis, CSF 5-HIAA and HVA levels did not differ significantly between individuals who died at a younger age and those who died later after adjustment for confounders. Although unadjusted analyses suggested lower metabolite levels in younger deceased patients, these differences were not statistically significant when controlling for demographic variables. No differences in CSF 5-HIAA were observed across causes of death, and no association was found between prior suicide attempts and either mortality or CSF monoamine levels. Overall, CSF 5-HIAA was not associated with early mortality or suicidal history in this cohort (76).
19	Cholesterol, CSF 5-HIAA, violence and intent in suicidal men	Jokinen J, Nordström AL, Nordström P.	The authors investigated associations among serum cholesterol, CSF 5-HIAA, suicide intent (planning subscale), and the violence of suicide attempt method in medication-free male individuals who attempted suicide compared with healthy controls. The study analyzed how these biological and clinical variables interrelated, with a focus on their relationship to dimensions of suicidal behavior, including intent and method.	10.1016/j.psychres.2008.0 7.020	2010	Individuals who attempted suicide showed significantly lower HDL-C levels compared with healthy controls, while total cholesterol and LDL-C did not differ, with results remaining significant after adjustment for age and BMI. Depression severity was inversely correlated with total cholesterol, and HDL-C declined with age. No differences in lipid levels were observed across suicide method or outcome groups. HDL-C was positively associated with the planning component of suicide intent in non-violent attempters. CSF 5-HIAA did not correlate with lipid measures in the overall sample, but in suicide victims it showed positive correlations with total cholesterol and LDL-C. Overall, serotonergic markers and lipid profiles were associated in a context- and subgroup-dependent manner, particularly in relation to suicide intent and outcomes (77).
20	Cholesterol and CSF 5-HIAA in attempted suicide	Asellus P, Nordström P, Jokinen J.	The authors examined the relationship between serum cholesterol and cerebrospinal fluid (CSF) 5-HIAA in individuals who attempted suicide, and to assess whether these measures are associated with suicidal behavior characteristics, including suicide lethality (violent methods), suicide intent, hopelessness, and depression severity.	10.1016/j.jad.2010.02.111 .	2010	The authors demonstrated a significant positive correlation between CSF 5-HIAA levels and serum total cholesterol in individuals who attempted suicide, which remained significant after adjusting for demographic and clinical covariates. Serum cholesterol was also associated with age and BMI. However, no significant differences in CSF 5-HIAA or cholesterol were observed between violent and non-violent suicide attempts, nor were these biomarkers associated with suicide intent, depressive severity, hopelessness, or subsequent suicide outcomes. Overall, CSF 5-HIAA was related to cholesterol levels but not to clinical characteristics or lethality of suicidal ideation (78).
21	Neurometabolic disorders: potentially treatable abnormalities in patients with treatment-refractory depression and suicidal behavior	Pan LA, Martin P, Zimmer T, Segreti AM, Kassiff S, McKain BW, Baca CA, Rengasamy M, Hyland K, Walano N, Steinfeld R, Hughes M, Dobrowolski SK, Pasquino M, Diler R, Perel J, Finegold DN, Peters DG, Naviaux RK, Brent DA, Vockley J.	The authors investigated whether metabolic abnormalities, particularly those affecting monoamine neurotransmitter synthesis, are underrecognized contributors to treatment-refractory depression.	10.1176/appi.ajp.2016.15 111500	2017	Patients with treatment-resistant depression exhibited a high prevalence of neurometabolic abnormalities (~two-thirds of cases), with cerebral folate deficiency being the most common (36%). This condition was characterized by reduced CSF 5-methyltetrahydrofolate despite normal peripheral folate levels. Additional metabolic disturbances included abnormalities in acylcarnitine metabolism, creatine synthesis deficits, and rare genetic variants. Individuals with these abnormalities showed higher depressive severity and suicidal ideation, with more than half reporting prior suicide attempts. Folinic acid supplementation in patients with cerebral folate deficiency was associated with significant reductions in depressive symptoms and a consistent, though not always statistically significant, decrease in suicidal ideation, with approximately half achieving a ≥50% improvement in depression severity (79).
22	Salivary 5-hydroxyindole acetic acid (5-HIAA) in drug-naïve patients with short-illness-duration first episode major depressive disorder	Cubala WJ, Landowski J, Chrzanowska A.	The aim of the authors was to investigate whether salivary 5-HIAA (s5-HIAA) levels in major depressive disorder (MDD), given the established role of central serotonergic dysfunction and the lack of prior data on salivary measurements. The study focused on evaluating whether salivary 5-HIAA could reflect serotonergic alterations in MDD by comparing levels between patients and healthy controls and examining its potential relationship with depressive status.	PMID: 25702305	2014	In this case–control study of drug-naïve first-episode MDD patients and matched healthy controls, salivary 5-HIAA levels measured at multiple standardized morning time points and analyzed via ELISA did not differ significantly between the overall patient group and controls. However, subgroup analyses showed significantly lower salivary 5-HIAA in non-melancholic MDD patients compared with controls, while no differences were observed in melancholic patients. Salivary 5-HIAA was not correlated with depression severity or illness duration. Overall, peripheral 5-HIAA levels were comparable between groups, with differences emerging only in specific depressive subtypes (80).
23	Gender-Related Differences in the Relationship between Homovanillic Acid in the Cerebrospinal Fluid and Clinical Symptoms in Suicide Attempters	Westling S, Ambrus L.	The authors tried to assess the relationship between cerebrospinal fluid (CSF) homovanillic acid (HVA) levels and clinical symptoms associated with increased suicide risk in individuals who had attempted suicide.	10.1159/000529803	2023	No significant differences in CSF HVA levels or clinical variables were observed between male and female participants. No associations were found between CSF HVA and depressive symptoms, overall psychopathology, or suicidal ideation in the full sample. In sex-stratified analyses, an inverse association between CSF HVA and anxiety severity was observed only in women, with lower HVA levels significantly correlated with higher anxiety scores after adjustment for confounders. No significant associations were identified in men. Overall, reduced dopaminergic activity showed a sex-specific association with anxiety but not with depression or suicidal ideation (81).

**Figure 1:**
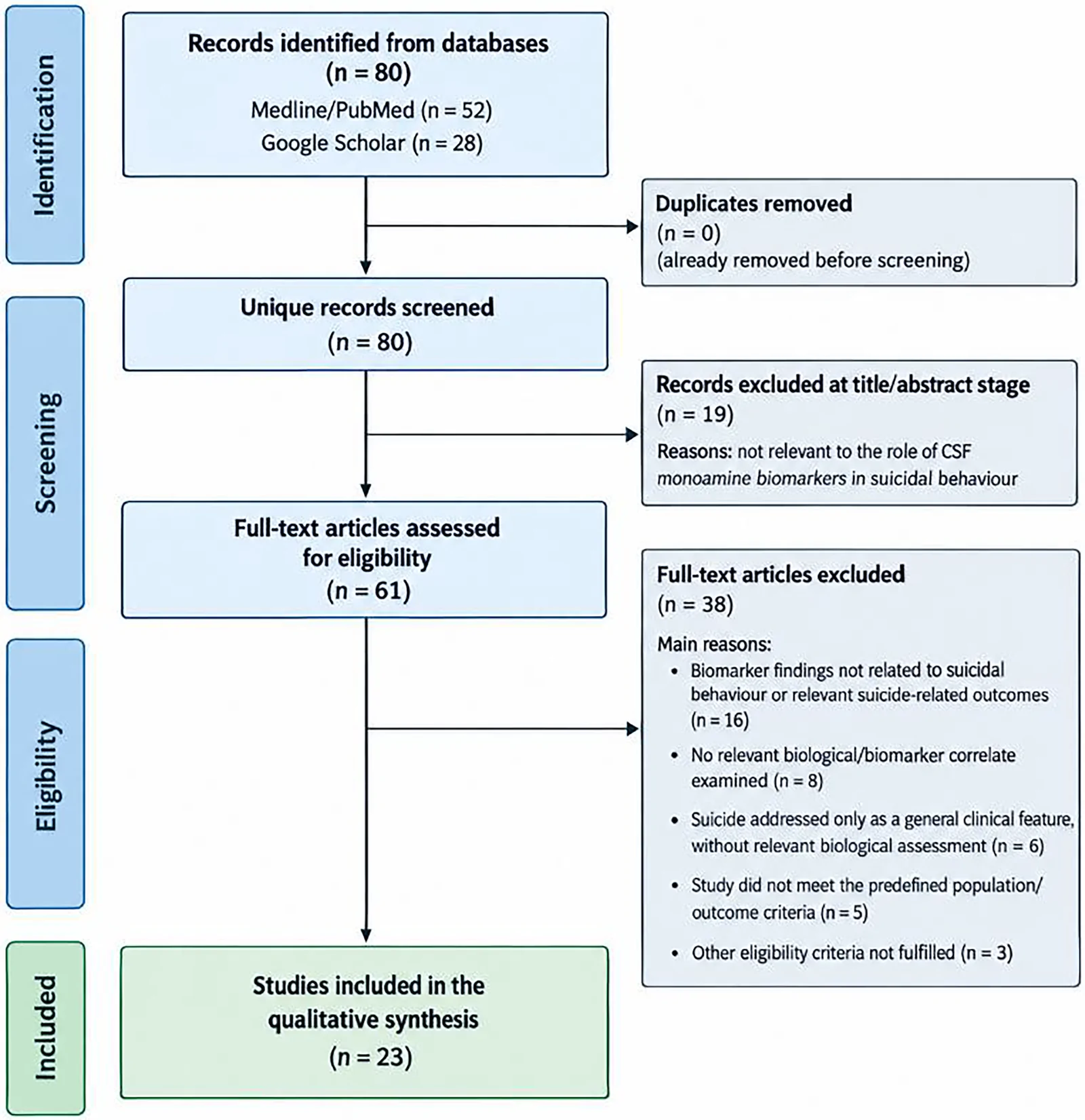
PRISMA-style flow diagram of the study-selection process: the diagram summarizes the identification, screening, eligibility assessment, and inclusion of studies in the narrative review. After removal of duplicate records, 80 unique records were screened; 19 were excluded at the title/abstract stage, 61 full-text articles were assessed for eligibility, and 38 were excluded following full-text assessment. A total of 23 studies were included in the qualitative synthesis.

The 23 studies retained for the review comprised primary biomarker studies directly assessing CSF 5-HIAA and/or HVA and a smaller group of contextual studies addressing other biological, clinical, demographic, genetic, or psychosocial correlates of suicidal ideation. The latter were retained to provide contextual information but were not considered equivalent to studies directly assessing CSF monoamine metabolites and were not used to draw conclusions regarding CSF 5-HIAA or HVA. Data were extracted from the included studies using a structured approach. The extracted information included study characteristics, study population, biological matrix, biomarkers investigated, analytical or methodological characteristics, and the main findings relevant to suicidal behavior, suicide attempts, lethality, or other suicide-related outcomes. No systematic reference-list screening or forward citation tracking was performed beyond the database searches described above. The findings from the included studies were synthesized narratively and organized according to the main biological and clinical aspects relevant to the review objectives. The synthesis considered the study population, type of suicidal behavior or outcome, biological matrix, biomarkers investigated, and the main associations reported between biomarker findings and suicidal ideation, suicide attempts, or lethality. No single validated methodological quality or risk-of-bias instrument applicable to all study designs was used. Given the heterogeneity of the included studies in terms of design, population, sample size, biological matrix, follow-up, and analytical methods, the findings were interpreted qualitatively and with consideration of the methodological characteristics and limitations of individual studies.

### 2.1 Inclusion criteria

Original studies published between 2005 and 2026, in English, were considered eligible if they investigated biological, neurobiological, biochemical, clinical, demographic, genetic, or psychosocial correlates of suicidal ideation, suicide attempts, or lethality, or other suicide-related outcomes, including studies in which these metabolites were assessed as part of a broader panel of biomarkers. No restriction was imposed *a priori* on a specific biomarker or biological pathway. Following study selection, the identified evidence was organized thematically according to the biomarker or biological domain investigated. CSF monoamine metabolites, particularly 5-HIAA and HVA, received greater attention because they represented the most extensively investigated biomarkers among the eligible studies. In this context, selected studies investigating 5-HIAA and/or HVA in other biological matrices, as well as studies examining closely related serotonergic, kynurenine, metabolic, inflammatory, or other neurobiological pathways, were retained when they provided relevant contextual evidence for the interpretation of suicidal behavior. Studies providing relevant clinical, demographic, genetic, or psychosocial context for suicidal ideation were also considered eligible when they contributed to the interpretation of the biological evidence or the factors associated with suicidal ideation.

### 2.2 Exclusion criteria

Publications in languages other than English; publications before 2005; review articles; studies that did not investigate biological, neurobiological, biochemical, clinical, demographic, genetic, or other relevant correlates of suicidal ideation, suicide attempts, lethality, or other predefined suicide-related outcomes; studies reporting biomarker values without relating them to suicidal ideation or other relevant suicide-related outcomes; studies focusing exclusively on biological findings without a defined contribution to the review question; and studies addressing suicide risk only in general terms, with psychiatric comorbidity as the principal focus and without examination of relevant biological or biomarker correlates. Studies assessing 5-HIAA and/or HVA in biological matrices other than CSF were excluded when they did not provide relevant contextual evidence for the interpretation of the biological findings.

### 2.3 Methodological quality and risk-of-bias assessment

The methodological quality and risk of bias of the 23 included studies were assessed using structured appraisal criteria appropriate to the study design. The assessment considered the appropriateness of the study population and participant selection, comparability of groups, validity and reliability of exposure and outcome measurements, measurement of biological markers, identification and management of potential confounding factors, completeness of follow-up where applicable, and the appropriateness of the statistical analyses. For longitudinal studies, particular attention was given to the temporal relationship between biomarker assessment and subsequent suicidal outcomes. Post-mortem studies were additionally considered in relation to the selection and comparability of cases and controls and potential post-mortem or tissue-related sources of bias. The genetic/computational study was evaluated separately according to the appropriateness of the genetic and statistical methodology and the validity of the analytical assumptions. The methodological appraisal was used to contextualize the interpretation of the findings and to distinguish more robust biomarker associations from findings potentially affected by small sample sizes, selection bias, confounding, measurement variability, or other methodological limitations. The assessment was not used as an exclusion criterion, given the heterogeneous observational designs and the narrative nature of the review (**Table 2**).

**Table 2:** Methodological quality and risk-of-bias assessment of the included studies.

Study	Design	Appraisal tool	Main methodological strengths	Main limitations	Overall appraisal
Sher et al.	Cohort/prospective	JBI Cohort	Prospective follow-up; defined biomarker assessment	Limited sample; clinical population	…
Chatzittofis et al.	Comparative/longitudinal	JBI	Biomarker + follow-up	Small sample; diagnostic heterogeneity	…
Bay-Richter et al.	Longitudinal	JBI Cohort	Repeated CSF measurements	Limited sample/generalizability	…
Cubala et al.	Case-control	JBI Case-Control	Matched controls; standardized sampling	Peripheral matrix; limited generalizability	…

The results of the methodological appraisal are summarized in **Table 1**. Overall, the included studies showed variable methodological quality.

The main limitations identified across the evidence base were small sample sizes, heterogeneous clinical populations, potential residual confounding, differences in biological sample collection and analytical procedures, and, in cross-sectional studies, limited ability to establish temporal relationships between biomarker levels and suicidal outcomes. These limitations were taken into account when interpreting the consistency and strength of the biomarker-related findings.

## 3 Results

The twenty-three studies considered in the review are summarized in **Table 1**. The primary evidence synthesis focused on studies directly assessing CSF 5-HIAA and/or HVA in relation to suicidal ideation, suicide attempts, lethality, or subsequent suicide-related outcomes. These studies were organized according to the main clinical and biological characteristics examined, including diagnostic groups, suicide-attempt lethality, and prospective outcomes. Selected studies addressing other biological matrices, biological pathways, or clinical and psychosocial correlates were considered separately as contextual evidence and were not treated as equivalent to the primary CSF biomarker evidence. The primary evidence synthesis focuses on studies directly investigating CSF 5-HIAA and HVA in relation to suicidal ideation, suicide attempts, lethality, or other suicide-related outcomes. Selected studies investigating 5-HIAA and/or HVA in other biological matrices, as well as studies examining closely related serotonergic, kynurenine, metabolic, inflammatory, or other neurobiological pathways, were retained as contextual evidence when relevant to the interpretation of suicidal behavior. Selected clinical, demographic, genetic, and psychosocial studies were also retained when they provided relevant contextual information for understanding suicidal ideation or interpreting the biological findings. These contextual studies are not considered equivalent to the primary CSF evidence and are not used to draw conclusions regarding CSF 5-HIAA or HVA concentrations. In addition, several *in vivo* and post-mortem investigations have explored potential biological correlates of suicidality, with particular attention to serotonergic and dopaminergic systems. Among the most extensively studied biomarkers are 5-hydroxyindoleacetic acid, the principal metabolite of serotonin, and homovanillic acid, a metabolite of dopamine. Evidence from cerebrospinal fluid studies in individuals with depressive disorders or a history of suicide attempts consistently indicates reduced levels of both 5-HIAA and HVA compared with healthy controls. Notably, lower concentrations of 5-HIAA have been associated with greater lethality of suicidal ideation, suggesting a potential relationship between serotonergic dysregulation and the severity of suicide attempts. Post-mortem studies have primarily focused on the quantification of serotonin (5-HT) and its metabolite 5-HIAA across different biological matrices. However, research specifically addressing the combined assessment of serotonergic and dopaminergic metabolites in post-mortem samples remains limited, with five studies reporting heterogeneous findings. The included studies varied in design and methodological approach. Their findings were synthesized narratively according to the main biological and clinical aspects relevant to the review objectives. Each article was reviewed in full, and the findings were synthesized narratively according to the main biological and clinical aspects relevant to the review objectives. The results were subsequently organized by different themes according to CSF 5-HIAA, CSF HVA, diagnostic groups, suicide-attempt lethality, and prospective suicide- related outcomes, while studies providing contextual evidence were considered separately.

### 3.1 Theme 1: CSF 5-HIAA and HVA: suicidal behavior and dopaminergic alterations

The primary CSF studies examined 5-HIAA and HVA in individuals with different psychiatric diagnoses and histories of suicidal behavior. The reported findings varied across diagnostic groups and study designs. Radenković et al. explored serotonergic system dynamics using a genotype-informed computational model integrating genetic variants involved in serotonin metabolism. In a heterogeneous psychiatric sample including mood and psychotic disorders, no significant association was found between modelled serotonergic features and suicide attempt history when considered independently. Instead, serotonergic dynamics, particularly those related to 5-HIAA, varied significantly across diagnostic categories. Interaction effects suggested that the relationship between serotonin system function and suicidality may be diagnosis-specific rather than universal. Overall, the study indicates that underlying psychiatric diagnosis exerts a stronger influence on serotonergic patterns than suicidal behavior alone, highlighting the complexity of disentangling biological correlates of suicidality (67). Neider et al. investigated long-term predictors of suicide in patients with schizophrenia, focusing on CSF monoamine metabolites and psychosocial factors. Over a follow-up period of approximately 25 years, suicide occurred in a subset of patients without significant differences in absolute CSF levels of HVA or 5-HIAA compared with survivors. However, the ratio between these metabolites (HVA/5-HIAA) was significantly lower in individuals who died by suicide, indicating a relative imbalance between dopaminergic and serotonergic systems. The HVA/5-HIAA ratio was significantly lower in individuals who died by suicide, whereas absolute CSF HVA and 5-HIAA levels did not differ significantly between groups. Exposure to childhood bullying was more frequent among those who died by suicide, suggesting an interaction between early-life stress and neurobiological vulnerability (70). Lindqvist et al. applied a multivariate biomarker approach to characterize neurobiological profiles associated with suicidal behavior. Using principal component analysis on a broad panel of CSF biomarkers, including monoamine metabolites, inflammatory markers, kynurenine metabolites, and neuropeptides, the authors identified distinct biological factors. One factor, characterized by high levels of monoamine metabolites (5-HIAA and HVA) and IL-6 and low levels of orexin, was significantly associated with violent suicide attempts and increased risk of suicide mortality during long-term follow-up. Another factor reflecting inflammatory activity was associated with higher suicidal intent. One factor characterized by higher levels of 5-HIAA and HVA, together with higher IL-6 and lower orexin levels, was associated with violent suicide attempts and increased risk of death by suicide during long-term follow-up (75). Westling et al. examined the relationship between dopaminergic activity and psychiatric symptoms in individuals who had recently attempted suicide. CSF levels of homovanillic acid were not associated with depressive severity or suicidal ideation. However, a significant sex-specific effect emerged: among female participants, lower CSF-HVA levels were independently associated with higher anxiety severity, even after adjusting for confounding variables. No such associations were observed in males. These findings indicate that reduced dopaminergic activity may contribute to anxiety symptoms in suicidal women, suggesting sex-dependent neurobiological mechanisms in suicidality (81). Moberg et al. (2011) investigated the relationship between exposure to interpersonal violence and CSF 5-HIAA levels in individuals who attempted suicide using structured psychiatric interviews, behavioral assessments, and biochemical analysis of CSF obtained under standardized conditions. The study applied correlation and subgroup analyses, including stratification by sex and trauma exposure. While CSF 5-HIAA levels did not differ across the full sample when stratified by demographic variables, a significant sex-specific association emerged: in women, higher exposure to childhood violence was associated with lower CSF 5-HIAA levels, an effect not observed in men. Further analyses showed that trauma exposure moderated the relationship between serotonergic activity and behavioral outcomes, with lower CSF 5-HIAA linked to increased expression of violent behavior in adulthood among individuals with high trauma exposure. These associations were robust after adjustment for relevant covariates such as age, sex, and depression severity. Overall, the findings indicate that serotonergic functioning may interact with early-life environmental stressors in shaping behavioral dysregulation relevant to suicidal and aggressive tendencies (74).

Bach et al. reported region-specific serotonergic alterations in postmortem brain tissue from suicide cases. Increased absolute levels of serotonin and 5-HIAA were observed in the brainstem, accompanied by a reduced 5-HIAA/5-HT ratio, indicating decreased serotonergic turnover. In contrast, no significant differences were found in prefrontal cortex measures. The results suggest that suicides are characterized not by a global reduction in serotonin or 5-HIAA, but by altered serotonergic regulation and turnover across brain regions (72).

Asellus et al. (2010) observed a significant positive correlation between CSF 5-HIAA and serum total cholesterol in individuals who attempted suicide, independent of demographic and clinical covariates. However, neither CSF 5-HIAA nor cholesterol levels were associated with suicide attempt lethality (violent vs. non-violent), suicide intent, depressive severity, hopelessness, or subsequent suicide outcomes. Overall, the study suggests a link between serotonergic activity and cholesterol metabolism, without a clear relationship to clinical features or lethality of suicidal behavior (71).

### 3.2 Theme 2: diagnostic groups and clinical populations

The included CSF studies involved heterogeneous clinical populations, including patients with major depressive disorder, bipolar disorder, schizophrenia spectrum disorders, alcohol dependence, and mixed psychiatric samples. Findings for CSF 5-HIAA and HVA differed across diagnostic groups and clinical characteristics. In patients with major depressive disorder, alterations in CSF 5-HIAA were reported in association with suicidal ideation and, in some studies, with greater lethality or subsequent suicide-related outcomes. However, these findings were not consistent across all clinical populations. In bipolar depression, baseline CSF 5-HIAA, HVA, and MHPG levels were not associated with the subsequent occurrence of suicide attempts, although lower concentrations were associated with greater lethality among attempts occurring during follow-up. In schizophrenia spectrum disorders, long-term studies similarly provided mixed evidence, with no consistent association between absolute CSF 5-HIAA or HVA levels and subsequent suicide or mortality outcomes, although relative abnormalities between monoamine metabolites were observed in some analyses. Studies involving patients with alcohol dependence or mixed psychiatric populations further support the importance of diagnostic context. In depressed patients with comorbid alcohol dependence, lower CSF 5-HIAA was associated with higher-lethality suicide attempts, whereas studies including heterogeneous mood and psychotic disorders suggested that serotonergic measures may vary according to psychiatric diagnosis rather than suicidal ideation alone. Sex-specific findings reported in some populations, particularly for CSF HVA, further indicate that clinical and demographic characteristics may modify the relationship between monoaminergic biomarkers and suicidal behavior. Overall, the available evidence does not support a single diagnostic-independent pattern of CSF 5-HIAA or HVA alterations. Instead, the associations appear to be influenced by the underlying psychiatric disorder, comorbidities, and other clinical characteristics. These findings support the interpretation of CSF monoamine biomarkers within their specific diagnostic and clinical context rather than as universal biological markers of suicidal behavior. Pan et al. demonstrated that neurometabolic abnormalities are highly prevalent in individuals with treatment-refractory depression and may contribute to both depressive severity and suicidality. In a case–control study, approximately two-thirds of patients exhibited metabolic abnormalities, with cerebral folate deficiency (CFD) being the most common. This condition, characterized by reduced CSF 5-methyltetrahydrofolate despite normal peripheral levels, may impair monoamine synthesis pathways. Targeted treatment with folinic acid resulted in significant reductions in depressive symptoms and a consistent, though not always statistically significant, decrease in suicidal ideation (79).

### 3.3 Theme 3: CSF biomarkers and suicide-attempt lethality

Sher et al. (2007) demonstrated that, in a cohort of depressed patients with comorbid alcohol dependence, individuals who attempted suicide were stratified according to lethality of attempts (high vs. low) and compared on clinical and CSF monoamine metabolite levels. CSF was obtained via lumbar puncture and analyzed for 5-HIAA, HVA, and MHPG. While demographic and most clinical variables, including depression severity, suicidal ideation, and impulsivity, did not differ between groups, individuals with high-lethality suicide attempts exhibited significantly lower CSF 5-HIAA levels compared with low-lethality attempters. This reduction remained significant after adjustment for age and sex. No group differences were observed for CSF HVA or MHPG. Additionally, monoamine metabolite levels did not correlate with clinical measures of depression or suicidal ideation. Overall, the findings indicated that reduced serotonergic activity, as reflected by CSF 5-HIAA, was specifically associated with greater lethality of suicidal behavior rather than with the presence or severity of depressive symptoms (82). Sher et al. (2006) conducted a prospective longitudinal study in bipolar depressed patients, assessing baseline CSF monoamine metabolites and following participants for up to two years to evaluate subsequent suicidal behavior. CSF samples were collected at baseline and analyzed using standardized biochemical methods, while clinical and behavioral characteristics were assessed using structured instruments. Baseline comparisons showed no significant differences in monoamine metabolite levels between individuals who later attempted suicide and those who did not, nor were these metabolites associated with baseline depression severity. However, follow-up analyses revealed that lower baseline levels of CSF 5-HIAA, HVA, and MHPG were significantly associated with higher lethality of suicide attempts occurring during the follow-up period. These associations were independent of the occurrence of suicide attempts themselves. Clinical variables such as aggression, hostility, and smoking severity were not correlated with CSF metabolite levels. The results suggest that central monoaminergic activity is more closely linked to the medical severity of suicidal behavior than to its incidence (38).

Jokinen et al. (2010) analyzed associations among serum lipid profiles, CSF 5-HIAA, and characteristics of suicidal behavior in male individuals who attempted suicide and matched controls. CSF samples were obtained via lumbar puncture and analyzed alongside serum lipid measurements, while clinical assessments included standardized scales for depression severity and suicide intent. Compared with controls, individuals with a history of suicide attempt(s) showed significantly lower HDL cholesterol, whereas total cholesterol and LDL cholesterol did not differ significantly. Depression severity was inversely correlated with total cholesterol, and HDL cholesterol declined with age across the sample. CSF 5-HIAA levels did not show significant correlations with lipid measures in the overall sample; however, in subgroup analyses of suicide victims, positive associations emerged between CSF 5-HIAA and total cholesterol as well as LDL cholesterol. Additionally, HDL cholesterol correlated with the planning component of suicide intent in non-violent attempters. These findings suggest that metabolic and serotonergic markers may relate differently to distinct dimensions of suicidal behavior, particularly intent and method, rather than uniformly reflecting suicidal status (77).

Asellus et al. (2018) investigated CSF apolipoprotein E (ApoE) in individuals who attempted suicide and found no association with depression severity or history of previous attempts. Lower CSF ApoE levels were, however, significantly associated with less reversible (more lethal) suicide attempt methods, and a trend toward lower levels was observed in violent attempts. No correlation was found between CSF ApoE and CSF 5-HIAA, suggesting independent biological pathways. These findings support a potential link between ApoE and lethality, without direct involvement of serotonergic markers (78).

### 3.4 Theme 4: prospective and longitudinal evidence

Neider et al. investigated long-term predictors of suicide in patients with schizophrenia, focusing on CSF monoamine metabolites and psychosocial factors. Over a follow-up period of approximately 25 years, suicide occurred in a subset of patients without significant differences in absolute CSF levels of HVA or 5-HIAA compared with survivors. However, the ratio between these metabolites (HVA/5-HIAA) was significantly lower in individuals who died by suicide, indicating a relative imbalance between dopaminergic and serotonergic systems. This neurochemical imbalance, rather than isolated alterations, may reflect impaired regulation of impulsivity and affect. Additionally, exposure to childhood bullying was more frequent among those who died by suicide, suggesting an interaction between early-life stress and neurobiological vulnerability (70).

Lindqvist et al. used a multivariate biomarker approach to characterize neurobiological profiles associated with suicidal behavior. Using principal component analysis of a broad panel of CSF biomarkers, including monoamine metabolites, inflammatory markers, kynurenine metabolites, and neuropeptides, the authors identified distinct biological factors. One factor, characterized by higher levels of the monoamine metabolites 5-HIAA and HVA, together with higher IL-6 and lower orexin levels, was significantly associated with violent suicide attempts and an increased risk of suicide mortality during long-term follow-up. Another factor reflecting inflammatory activity was associated with higher suicidal intent. These findings highlight the heterogeneity of the biological correlates of suicidal behavior and indicate that monoaminergic alterations may not follow a uniformly low pattern. Rather, their associations with suicidality may depend on interactions with inflammatory and neuroendocrine systems, as well as on the clinical characteristics and severity of suicidal behavior (75).

Sher et al. (2006) conducted a prospective longitudinal study in patients with bipolar depression, measuring CSF 5-HIAA, HVA, and MHPG at baseline and evaluating suicidal behavior over a two-year follow-up period. Baseline metabolite levels did not differ significantly between patients who subsequently attempted suicide and those who did not, and were not associated with baseline depression severity. However, lower baseline concentrations of 5-HIAA, HVA, and MHPG were significantly associated with greater lethality of suicide attempts occurring during follow-up. Lower baseline concentrations of 5-HIAA, HVA, and MHPG were significantly associated with greater lethality of suicide attempts during follow-up, whereas baseline levels did not differ between patients who subsequently attempted suicide and those who did not (38). Individuals who attempted suicide showed higher CSF and plasma cortisol levels compared with controls, indicating HPA axis hyperactivity, while CSF DHEAS did not differ overall except for higher levels in males. CSF 5-HIAA levels were reduced in attempters, with significant differences observed in females. Correlations indicated relationships among cortisol, DHEAS, and a trend with 5-HIAA. Childhood violence exposure was associated with altered stress markers, including a lower cortisol/DHEAS ratio. Follow-up suggested that individuals who committed suicide tended to have lower CSF 5-HIAA and higher cortisol levels, supporting a link between serotonergic and HPA axis dysregulation and suicidal outcomes (73).

Carlborg et al. examined CSF monoamine metabolites in a long-term cohort of patients with schizophrenia spectrum psychosis and found no significant association between CSF 5-HIAA levels and early mortality after adjustment for confounders. CSF 5-HIAA did not differ across causes of death, nor was it associated with prior suicide attempts. These findings indicate that CSF 5-HIAA is not a reliable predictor of mortality or suicidal history in this population (76).

### 3.5 Theme 5: contextual evidence on clinical, genetic, psychosocial, and other biological correlates of suicidal behavior

A subset of studies provided contextual evidence on clinical, demographic, genetic, psychosocial, or other biological factors associated with suicidal behavior. These studies did not directly investigate CSF 5-HIAA and/or HVA and were therefore considered separately from the primary CSF biomarker evidence. Their findings are summarized here only to provide contextual information for the interpretation of suicidal behavior and are not used to draw conclusions regarding CSF 5-HIAA or HVA. Laskowski et al. reported associations between lifetime suicide attempts and several sociodemographic and psychosocial characteristics among adult men, including education, employment, relationship status, and migration background (61).

Zhang et al. reported an association between suicidal intent and the lethality of the method used in suicide attempts, with interpersonal conflict and economic difficulties among the reported precipitating factors (62).

Bitar et al. identified different adolescent health-behavior profiles and reported higher prevalence of suicidal ideation and attempts in profiles characterized by multiple co-occurring risk behaviors (63).

Chen et al. investigated trajectories of cumulative life-event exposure and suicidal ideation during adolescence. Four trajectories of life-event exposure were identified, and adolescents belonging to the persistently high-exposure trajectory showed higher and more stable levels of suicidal ideation over time. This association remained significant after adjustment for sociodemographic and adverse childhood factors. Family-related stressors were among the factors associated with suicidal ideation (64). Yao et al. examined the genetic architecture of suicide-related traits using genomic structural equation modeling and post-GWAS analyses. The study identified a latent genetic factor encompassing suicidal behavior, depression, loneliness, childhood trauma, financial hardship, suicidal ideation, and chronic pain. Genome-wide analyses identified multiple associated loci and genes involved in processes including protein homeostasis, synaptic function, intracellular trafficking, and DNA repair (65). Seedat et al. examined cross-national gender differences in the prevalence of mental disorders using data from the World Mental Health Surveys. Women showed higher lifetime prevalence of internalizing disorders, particularly mood and anxiety disorders, whereas men showed higher prevalence of externalizing and substance-related disorders. The magnitude of these differences varied across disorders, countries, and birth cohorts (66). By-Richter et al. identified persistent dysregulation of the kynurenine pathway in individuals with a history of suicide attempt, suggesting a role of immune–metabolic mechanisms in suicidality. In a longitudinal design with repeated CSF sampling, patients exhibited elevated levels of quinolinic acid (QUIN), a neurotoxic NMDA receptor agonist, and reduced levels of kynurenic acid (KYNA), a neuroprotective NMDA antagonist, compared with healthy controls. Importantly, KYNA levels were inversely associated with both depressive symptom severity and suicidal ideation over time, whereas QUIN showed no consistent relationship with clinical measures. Additionally, interleukin-6 (IL-6) levels were positively correlated with suicidal symptom severity, supporting the involvement of inflammatory processes. These findings suggest a sustained shift toward excitotoxicity and reduced neuroprotection in suicidal individuals (69). Pan et al. demonstrated that neurometabolic abnormalities are highly prevalent in individuals with treatment-refractory depression and may contribute to both depressive severity and suicidality. In a case–control study, approximately two-thirds of patients exhibited metabolic abnormalities, with cerebral folate deficiency (CFD) being the most common. This condition, characterized by reduced CSF 5-methyltetrahydrofolate despite normal peripheral levels, may impair monoamine synthesis pathways. Targeted treatment with folinic acid resulted in significant reductions in depressive symptoms and a consistent, though not always statistically significant, decrease in suicidal ideation. These findings suggest that metabolic dysfunctions represent a potentially modifiable biological substrate underlying treatment resistance and suicidal behavior (79). Cubala et al. (2014) evaluated salivary 5-HIAA levels in drug-naïve patients with first-episode major depressive disorder (MDD) compared with healthy controls using a cross-sectional case-control design. Saliva samples were collected at multiple standardized time points in the morning and analyzed using enzyme-linked immunosorbent assay (ELISA), with mean values used for statistical analysis. Clinical diagnoses were established using structured interviews, and depression severity was quantified using standardized rating scales. Overall, no significant differences in salivary 5-HIAA levels were observed between the full MDD group and controls.

However, subgroup analyses revealed lower 5-HIAA levels in patients with non-melancholic depression compared with controls, whereas melancholic patients did not differ from controls. Furthermore, salivary 5-HIAA levels were not correlated with depression severity or illness duration. These results suggest that peripheral serotonergic metabolites may vary according to depressive subtype rather than reflecting global depression severity (80). Pompili et al. demonstrated that, in hospitalized patients with major depressive and bipolar disorders, plasma tryptophan and kynurenine metabolism was significantly altered compared with healthy controls, with higher tryptophan, lower kynurenine, and an increased TRY/KYN ratio. However, no significant differences were observed for 5-HIAA or other kynurenine pathway metabolites between groups, and 5-HIAA levels were not associated with a history of suicide attempts. Overall, serotonergic turnover indices did not show a consistent relationship with suicidal ideation in this sample (68).

## 4 Discussion

Individuals who engage in high-lethality suicide attempts, which closely resemble to completed suicides in terms of behavioral characteristics, may also share similar biochemical profiles. Research into the neurobiology of high-lethality suicide attempts has the potential to elucidate the underlying pathophysiological mechanisms of suicide. Furthermore, such studies could aid in the identification of biological markers predictive of suicide risk and reveal potential therapeutic targets for interventions aimed at reducing suicidal ideation. Identifying reliable biological predictors of high-lethality suicide attempts would be of considerable value for improving suicide risk assessment and prevention strategies (83). Importantly, statistically significant differences between groups should not be interpreted as evidence of predictive performance or clinical utility. Although the reported biomarkers may be associated with the investigated clinical conditions, their potential value for prediction or clinical risk stratification requires assessment through appropriate predictive-performance measures and independent validation. Therefore, the available findings should be considered preliminary and hypothesis-generating rather than sufficient to support clinical application. An important consideration is that suicidal ideation, suicide attempts, non-suicidal self-injury, self-harm, and suicide death represent distinct clinical and behavioral outcomes and should not be considered interchangeable. These phenomena may differ in terms of suicidal intent, severity, lethality, measurement, and underlying mechanisms. Similarly, suicide attempts may vary substantially in intent and medical severity, including high-lethality attempts, and should therefore not necessarily be interpreted as a homogeneous outcome. Accordingly, findings involving these outcomes should be interpreted in relation to the specific phenotype assessed in each study rather than generalized across the broader spectrum of suicidal and self-harm behaviors. Chen et al. published a review highlighting converging evidence pointing out that structural brain alterations in individuals with major depressive disorder (MDD) and suicide attempts (SA) predominantly involve fronto-parietal-temporal-limbic circuitry, suggesting a shared neurobiological substrate of suicidality across the lifespan. Despite this overall consistency, age-dependent patterns of brain abnormalities emerge, indicating that the neuroanatomical correlates of suicidal ideation are partially modulated by developmental stage. Across all age groups, the prefrontal cortex (PFC), particularly the ventrolateral (VLPFC) and orbitofrontal cortex (OFC), appears critically implicated. These regions are central to emotional regulation, decision-making, and processing of reward and punishment signals, and their structural abnormalities may contribute to impaired evaluation of negative outcomes and reduced behavioral inhibition, both of which are associated with suicidal vulnerability. Consistently, reductions in grey matter volume (GMV) in these regions were observed in suicidal individuals, supporting their role as potential neurobiological markers of suicide risk. The dorsolateral prefrontal cortex (DLPFC), involved in executive control and emotional regulation, also shows significant volume reductions, particularly in middle-aged patients. Notably, neuromodulation targeting this region (e.g., transcranial magnetic stimulation) has been associated with reductions in suicidal ideation, reinforcing its functional relevance. However, such alterations were less consistently observed in elderly populations, possibly due to age-related neuropathological complexity (e.g., cerebrovascular changes or neurodegeneration), which may obscure or interact with depression-related structural changes. Alterations in the limbic system, including the amygdala, hippocampus, and basal ganglia, further support the involvement of emotional processing and stress-response systems in suicidality. In particular, changes in the striatum and cingulate cortex suggest dysregulation of reward processing and affective integration. Additionally, evidence linking suicidality to serotonergic dysfunction, especially within ventral PFC regions, aligns with structural findings and supports a neurochemical–structural interaction underlying suicidal ideation. The angular gyrus emerges as another region of interest, though with age-specific patterns: decreased GMV in middle-aged individuals versus increased GMV in adolescents. This divergence may reflect differences in cognitive-emotional processing and environmental stressors, with adolescents being more influenced by interpersonal and developmental factors, whereas adults are more affected by somatic illness and socioeconomic stress. A further age-related distinction concerns cortical thickness. While middle-aged and elderly individuals with SA tend to show accelerated cortical thinning, adolescents exhibit atypical increases in cortical thickness, possibly reflecting disrupted neurodevelopmental trajectories. Given that adolescence is characterized by dynamic cortical remodeling, these findings may indicate delayed or aberrant maturation processes associated with suicide risk. Overall, the findings support a model in which suicidal ideation in MDD arises from dysfunction within a distributed neural network integrating executive control, emotional regulation, and reward processing, with both shared and age-specific neurobiological features. These results underscore the importance of incorporating age as a key variable in neuroimaging studies of suicidality and suggest that structural brain markers may contribute to improved risk stratification and targeted interventions (84). A substantial body of postmortem and neurobiological evidence indicates that suicidal behavior is associated with a dysregulation of the serotonergic (5-HT) system, involving both central neuroanatomical abnormalities and measurable biochemical changes in CSF. In particular, the findings support a model of reduced serotonergic neurotransmission accompanied by compensatory neurobiological adaptations. At the level of the brainstem, particularly within the dorsal raphe nucleus (DRN), suicides exhibit increased density and proportion of serotonergic neurons, as well as upregulated expression of tryptophan hydroxylase 2 (TPH2), the rate-limiting enzyme in 5-HT synthesis. Despite this apparent increase in serotonergic cellular markers, multiple lines of evidence, including reduced cerebrospinal fluid 5-HIAA levels, indicate a functional deficit in serotonin transmission. This apparent paradox suggests that the observed upregulation reflects a compensatory response to impaired serotonin release or reduced synaptic availability, rather than enhanced serotonergic function. At the cortical level, particularly in the prefrontal cortex (PFC), suicides show reduced serotonin transporter (SERT) binding, consistent with decreased serotonergic innervation or altered synaptic reuptake mechanisms. This reduction appears to be specifically associated with suicidal ideation, independent of psychiatric diagnosis in some cases, and is particularly relevant in regions implicated in behavioral inhibition and emotional regulation. Alterations in serotonin receptor systems further support the presence of compensatory mechanisms. Increased binding of postsynaptic 5-HT1A and 5-HT2A receptors in the PFC has been frequently reported, likely reflecting upregulation in response to chronically low serotonin levels. In contrast, changes in presynaptic 5-HT1A auto-receptors within the raphe nuclei may enhance inhibitory feedback on serotonergic neurons, further contributing to reduced serotonin release. This imbalance between pre- and postsynaptic regulation may play a critical role in the pathophysiology of suicidality. Importantly, serotonergic dysfunction appears to involve region-specific and circuit-level abnormalities, particularly within pathways connecting the raphe nuclei to limbic and prefrontal regions, which are central to stress response, emotional processing, and impulse control. The involvement of additional modulatory systems, including GABAergic interneurons within the raphe, suggests a complex regulatory network influencing serotonergic output. Taken together, these findings support a model in which suicidal ideation is associated with a hypofunctional serotonergic state, characterized by impaired neurotransmission despite molecular and cellular upregulation of serotonergic components. However, it remains unclear whether these alterations represent a primary causal mechanism or a secondary adaptive response to underlying neurobiological or environmental stressors. In parallel, alterations in dopaminergic metabolism have also been described, with changes in CSF levels of HVA, the main dopamine metabolite. Although findings on HVA are less consistent compared with 5-HIAA, some studies suggest that reduced HVA levels may reflect impaired dopaminergic activity, particularly in circuits involved in reward processing, motivation, and impulsivity, which are known to contribute to suicidal ideation.

The combined reduction of 5-HIAA and HVA may therefore indicate a broader dysregulation of monoaminergic systems, rather than an isolated serotonergic deficit (85). Sex differences have been consistently observed in serotonin-related psychiatric conditions, including depression, anxiety, and suicidal ideation. Neuroimaging evidence from positron emission tomography (PET) studies indicates that women exhibit higher binding potentials of 5-HT1A receptors and lower binding of the serotonin transporter (5-HTT) across a broad range of cortical and subcortical regions compared with men. These findings suggest underlying biological differences in the serotonergic system that may contribute to sex-specific patterns in the prevalence and expression of psychiatric disorders (86). This evidence suggests that abnormalities in central neurotransmitter systems, particularly serotonergic and dopaminergic pathways, may contribute to vulnerability to suicidal ideation; reduced levels of 5-HIAA, are associated with impaired regulation of aggressive behavior in adulthood. Such dysregulation, in conjunction with impulsivity and aggressiveness, has been proposed as an endophenotypic trait underlying both suicidal and violent behaviors, thereby acting as a potential mediator of increased risk for suicide and violence (74). Substantial evidence indicates that cerebrospinal fluid levels of 5-HIAA are significantly reduced in individuals who attempt suicide compared with psychiatric controls, supporting a central role of the serotonergic system in suicidality. In contrast, findings related to dopaminergic and noradrenergic metabolites, such as HVA and MHPG, remain inconsistent. Notably, the association between reduced CSF 5-HIAA levels and suicidal behavior appears to be particularly pronounced in individuals engaging in violent suicide attempts, suggesting that serotonergic dysfunction may be especially relevant in more severe or high-lethality forms of suicidal ideation. This pattern reinforces the hypothesis that impaired serotonergic activity contributes to the regulation of impulsivity and aggression, key components underlying suicidal acts. Importantly, reduced 5-HIAA levels have been observed across a wide range of psychiatric conditions, including mood disorders, personality disorders, substance use disorders, and schizophrenia, indicating that serotonergic dysfunction represents a transdiagnostic feature of suicidal ideation rather than a disorder-specific abnormality. The available evidence suggests that lower CSF 5-HIAA may be associated with specific aspects of suicidal ideation particularly greater lethality of suicide attempts and, in some studies, specific diagnostic or clinical subgroups. However, these associations should not be interpreted as evidence of a general predictive capacity for future suicidal ideation. Prospective studies have yielded mixed findings, with baseline CSF 5-HIAA not consistently distinguishing individuals who subsequently attempted suicide from those who did not. Thus, although CSF 5-HIAA may represent a relevant biological correlate of suicidal behavior and its severity in selected contexts, current evidence is insufficient to support its use as a validated predictor or clinically applicable biomarker of future suicidal ideation. These findings support the potential relevance of serotonergic alterations to suicidal ideation, while underscoring the need for further prospective and mechanistic studies before their clinical significance can be established (60). Although the association between reduced CSF 5-HIAA concentrations and suicidal behavior is consistent with a possible involvement of serotonergic pathways, these findings should not be interpreted as providing direct evidence suggesting that the increased serotonergic neurotransmission prevents suicidal ideation. The clinical effects of SSRIs are complex and may vary according to diagnosis, age, treatment phase, and other patient- and treatment-related factors. Thus, the observed neurobiological associations may provide a basis for further investigation of serotonergic mechanisms, but they do not by themselves establish a preventive or therapeutic effect of SSRIs on suicidal ideation. Specifically, the meta-analytic results published by Hoertel et al., indicate that individuals who attempted suicide exhibit significantly reduced CSF levels of 5-HIAA, supporting a state of central serotonin hypofunction, which appears consistent across both violent and non-violent attempts and largely independent of demographic or diagnostic variables. These findings are coherent with post-mortem data showing widespread alterations within the dorsal raphe nucleus, including increased density of serotonergic neurons and upregulation of TPH2 expression. This apparent discrepancy, reduced serotonin metabolite levels alongside increased serotonergic neuronal markers, suggests the presence of a compensatory upregulation in response to impaired serotonin release or reduced synaptic availability. Overall, this pattern supports the hypothesis that suicidal ideation is characterized not merely by a deficit in serotonin synthesis, but by a more complex dysregulation of serotonergic neurotransmission involving impaired release, altered receptor function, and compensatory neuroadaptive mechanisms (59).

Importantly, the association between CSF monoamine metabolites and suicidal ideation appears to vary across psychiatric diagnoses. In contrast to the consistent findings observed in mood disorders, evidence from schizophrenia spectrum psychosis remains inconclusive. A large longitudinal study with a 24-year follow-up found no significant differences in CSF levels of 5-HIAA, HVA, or their ratio between individuals who died by suicide, attempted suicide, or did not exhibit suicidal ideation. Moreover, CSF 5-HIAA levels were not predictive of future suicide risk, even when stratified by lethality or follow-up duration, and no association was observed with violent versus non-violent suicide. Instead, clinical variables, particularly prior suicide attempts (OR ≈ 12.4) and male sex (OR ≈3.5), emerged as the strongest predictors of suicide mortality, highlighting the predominant role of established clinical risk factors over biological markers in this population. These findings suggest that, unlike in affective disorders, serotonergic dysfunction as reflected by CSF 5-HIAA may not represent a reliable biomarker of suicide risk in schizophrenia spectrum disorders. This discrepancy supports the hypothesis that the neurobiological underpinnings of suicidality are, at least in part, diagnosis-specific, with serotonergic abnormalities playing a more central role in mood-related psychopathology.

Furthermore, it underscores the potential influence of confounding factors specific to schizophrenia, such as antipsychotic treatment effects, illness chronicity, and psychotic symptomatology, which may obscure or modulate underlying monoaminergic abnormalities. Overall, these results highlight the need for a more stratified, diagnosis-sensitive approach in the identification and interpretation of biological markers of suicide risk (87). From a broader theoretical perspective, classical conceptualizations of suicidal ideation, including those proposed by Durkheim, Freud, and Shneidman, have emphasized the heterogeneity of suicidal acts and their underlying psychological and social dimensions. Although these frameworks were not developed in relation to biological biomarkers, their emphasis on different forms and functions of suicidal behavior may provide a conceptual context for considering whether distinct biological profiles could accompany different suicidal phenotypes. In this regard, the monoaminergic findings reviewed here could be viewed as generating hypotheses about possible biological heterogeneity across different manifestations of suicidal ideation. However, these potential relationships remain speculative, as the studies included in this review did not directly test associations between the classical typologies and specific monoaminergic endophenotypes. Further studies integrating clinical phenotyping with biological measures would therefore be required to determine whether such theoretical distinctions correspond to reproducible neurobiological profiles (54, 88, 89).

Several methodological limitations of the included studies should be acknowledged, as they affect the interpretation and generalizability of the reported findings. First, the majority of CSF studies in suicidal populations are based on relatively small sample sizes, which limits statistical power and increases the risk of both false-positive and false-negative results. As noted by Hoertel et al. in their meta-analysis, most individual studies relied on limited numbers of participants, and the overall quality of evidence remains constrained by this factor (59). Second, substantial heterogeneity exists in CSF collection and processing protocols across studies, including differences in the timing of lumbar puncture, patient positioning, volume of CSF extracted, storage conditions, and assay methodology (90). Such variability may introduce pre-analytical and analytical confounders that complicate cross-study comparisons of monoamine metabolite concentrations. Third, the diagnostic heterogeneity of study populations represents a further limitation. The included studies enrolled patients with mood disorders, schizophrenia spectrum disorders, personality disorders, and substance use disorders, often without stratification by diagnosis. Given that the association between CSF 5-HIAA and suicidal behavior appears to be diagnosis-dependent, as illustrated by the negative findings in schizophrenia cohorts, pooling results across diagnostic categories may obscure clinically meaningful differences (60). Fourth, most studies were cross-sectional or included only a single CSF sampling point, precluding assessment of intra-individual variability in monoamine metabolite levels over time. The few longitudinal designs with repeated CSF sampling, such as that by Bay-Richter et al., remain exceptions The methodological appraisal further highlighted that the available evidence is heterogeneous in design and methodological robustness, and that findings from individual studies should therefore be interpreted in the context of sample size, population characteristics, potential confounding, biomarker measurement procedures, and study design limitations. From a translational perspective, the clinical applicability of CSF monoamine metabolites as routine biomarkers for suicide risk assessment faces significant practical barriers (69).

Moreover, we point out an additional limitation. No single validated risk-of-bias instrument applicable to all study designs was used. The included studies differed substantially in study design, population characteristics, sample size, biological matrix, follow-up duration, and analytical procedures. Consequently, the findings should not be considered equivalent in terms of evidentiary strength. The interpretation was therefore qualitative, with greater emphasis placed on findings supported by controlled or comparative human studies, while observational, preclinical, and other lower-level evidence was considered more cautiously. This methodological heterogeneity limits the comparability of individual findings and should be taken into account when interpreting the potential relevance of CSF 5-HIAA and HVA as biological correlates of suicidal ideation. In addition to the limitations of the individual studies, several limitations of the present review should be acknowledged. First, the literature search was conducted using PubMed and Google Scholar and was restricted to publications in English, which may have resulted in the omission of relevant studies indexed in other databases or published in other languages. Second, the predefined publication period (2005–2026) was selected to focus the primary synthesis on contemporary evidence and may have excluded earlier studies that could provide additional historical or scientific context. Third, the narrative design of the review did not allow quantitative pooling of effect estimates or formal assessment of publication bias. This was considered appropriate given the substantial heterogeneity in study populations, clinical diagnoses, biological matrices, CSF collection procedures, biomarker assays, and outcome definitions. Finally, although structured design-appropriate methodological appraisal criteria were applied, no single validated risk-of-bias instrument applicable to all study designs was used. These limitations should be considered when interpreting the overall consistency and generalizability of the evidence summarized in this review.

Lumbar puncture, while generally considered a safe procedure, is invasive and associated with potential complications, most commonly post-lumbar puncture headache, reported in approximately 10 and 25% of cases in large multicenter feasibility studies (91). The procedure requires trained personnel, appropriate clinical settings, and informed consent, making it impractical for widespread screening in acute psychiatric care or primary care contexts. Furthermore, the cost and logistical requirements associated with CSF collection, storage, and analysis using high-performance liquid chromatography limit its feasibility as a point-of-care tool (92). Importantly, CSF monoamine metabolite levels reflect cumulative central neurotransmitter turnover rather than providing real-time information about acute neurochemical states, which further constrains their utility for identifying individuals at imminent risk of suicidal ideation. These limitations have stimulated growing interest in peripheral biomarkers as less invasive alternatives to CSF-based measures. Among the most investigated peripheral indices are plasma 5-HIAA and platelet serotonin content. Spreux-Varoquaux et al. reported that plasma 5-HIAA was significantly lower in impulsive violent individuals who attempted suicide compared with non-impulsive attempters and healthy controls, a pattern that mirrors the CSF 5-HIAA findings in similar populations (93). However, the relationship between central and peripheral serotonergic indices is complex and not straightforward. Strawn et al. demonstrated through serial CSF and blood sampling that longitudinal intra-individual changes in plasma 5-HIAA correlate positively with CSF 5-HIAA fluctuations, suggesting that relative changes in peripheral 5-HIAA may reflect central serotonergic dynamics within the same individual over time. Critically, however, no significant cross-sectional interindividual correlation between absolute plasma and CSF 5-HIAA levels was observed, indicating that a single plasma measurement cannot be used to infer an individual’s absolute CSF 5-HIAA concentration (94). Importantly, these findings do not support a simple or unidirectional low-monoamine model of suicidal ideation. For example, Lindqvist et al. identified a biological component characterized by higher 5-HIAA and HVA, together with inflammatory and neuroendocrine markers, that was associated with violent suicide attempts and increased risk of suicide mortality. This finding suggests that the relationship between monoaminergic alterations and suicidal ideation may depend on interactions with other biological systems and on clinical characteristics such as the phenotype and severity of suicidal ideation. Platelet serotonin content has also been proposed as a peripheral marker of central serotonergic function, given that platelets share uptake, storage, and release mechanisms with serotonergic neurons. Giurgiuca et al. reported significantly lower platelet serotonin levels in bipolar I patients who had attempted suicide compared with non-attempters, with further reductions observed in high-lethality attempters, suggesting a graded relationship between peripheral serotonergic deficit and severity of suicidal ideation (95). Similarly, Pivac et al. found reduced platelet serotonin concentrations in suicidal patients with combat-related PTSD, regardless of diagnostic group, supporting the potential transdiagnostic utility of this measure (96). Salivary 5-HIAA has been explored as an even less invasive alternative; however, Cubalea et al. found no significant difference in salivary 5-HIAA between drug-naive patients with first-episode major depressive disorder and healthy controls, and its relationship to suicidal ideation remains undetermined (80). A comprehensive review by Kang et al. further underscored that peripheral biomarkers of suicidal ideation, including inflammatory markers (IL-2, IL-6, CRP), cholesterol, folate, and serotonin-related indices, show individually weak associations with suicidal outcomes, and that combinations of multiple peripheral markers rather than single analytes may be necessary to achieve clinically meaningful predictive accuracy (97). Overall, while CSF 5-HIAA remains the most extensively validated neurochemical marker associated with suicidal ideation, the practical constraints of lumbar puncture highlight the need for parallel development of peripheral biomarker panels. Future research should prioritize large-scale, prospective studies that simultaneously assess central and peripheral serotonergic indices in the same individuals, with standardized collection protocols and diagnostic stratification, to determine whether peripheral measures can serve as reliable proxies for CSF-based assessments in clinical suicide risk evaluation.

The interpretation of CSF monoamine metabolite concentrations is further complicated by several clinical, biological, and pre-analytical factors. Psychiatric diagnosis and current clinical state, medication exposure and washout, substance use, dietary and fasting status, circadian timing, age, sex, physical activity, body position, CSF sampling characteristics, including collection site and volume, as well as sample handling, storage conditions, and analytical methodology may contribute to variability in measured metabolite concentrations. These factors were not consistently reported across the included studies, limiting the extent to which their potential contribution to between-study differences could be assessed. This methodological heterogeneity should therefore, be considered when interpreting differences in CSF 5-HIAA and HVA concentrations across studies and clinical populations.

## 5 Conclusion

In conclusion, the available evidence indicates that CSF monoamine metabolites, particularly 5-HIAA and HVA, may be relevant biological correlates of suicidal behavior and, in selected contexts, of the severity or lethality of suicidal acts. However, the findings are heterogeneous across diagnostic groups, clinical phenotypes, and study designs, and prospective evidence does not consistently support their ability to predict future suicidal behavior. The small sample sizes, invasive nature of CSF sampling, and absence of validated predictive thresholds further limit their current clinical applicability. Therefore, CSF 5-HIAA and HVA should presently be regarded as research-level biological correlates rather than clinically actionable biomarkers. Further prospective studies integrating CSF measures with clinical, genetic, inflammatory, and other biological factors are needed to determine whether these markers may ultimately contribute to clinically useful models of suicide risk. The available evidence supports the relevance of investigating CSF monoamine metabolites, particularly 5-HIAA and, to a lesser extent, HVA, as potential biomarkers of suicidal ideation. While reduced CSF 5-HIAA levels consistently reflect a state of central serotonergic dysregulation, especially in mood disorders and in more severe forms of suicidal ideation, the heterogeneity of findings across diagnostic groups highlights the complexity of the underlying neurobiology. Nonetheless, the integration of biochemical, post-mortem, and clinical data suggests that these markers capture meaningful alterations in neurotransmission that may contribute to vulnerability to suicide. From a translational perspective, the identification and validation of such biomarkers could support the development of objective tools for risk stratification, complementing clinical assessment. Moreover, the invasive nature of lumbar puncture, together with the limited evidence for individual-level predictive performance, makes CSF biomarkers unsuitable for routine suicide-risk screening at present. Further longitudinal and independently validated studies are needed to determine whether CSF monoamine metabolites have clinically meaningful predictive value. Any future application to individual risk stratification would also require careful consideration of the potential consequences of false-positive and false-negative classifications, including inappropriate intervention or missed opportunities for clinical care.
